# Infection Process and Genome Assembly Provide Insights into the Pathogenic Mechanism of Destructive Mycoparasite *Calcarisporium cordycipiticola* with Host Specificity

**DOI:** 10.3390/jof7110918

**Published:** 2021-10-28

**Authors:** Qing Liu, Yanyan Xu, Xiaoling Zhang, Kuan Li, Xiao Li, Fen Wang, Fangxu Xu, Caihong Dong

**Affiliations:** 1 State Key Laboratory of Mycology, Institute of Microbiology, Chinese Academy of Sciences, Beijing 100101, China; liuqingfungi@gmail.com (Q.L.); xuyanyan@im.ac.cn (Y.X.); zhangxl@im.ac.cn (X.Z.); likuan@im.ac.cn (K.L.); lixmushroom@gmail.com (X.L.); wangfen@im.ac.cn (F.W.); 2 University of Chinese Academy of Sciences, Beijing 100049, China; 3 Experimental Teaching Center, Shenyang Normal University, Shenyang 110034, China; dzb@synu.edu.cn

**Keywords:** *Calcarisporium cordycipiticola*, *Cordyceps militaris*, white mildew disease, infection process, genome sequencing, host specificity, necrotrophy, mycotoxin

## Abstract

*Calcarisporium cordycipiticola* is the pathogen in the white mildew disease of *Cordyceps militaris*, one of the popular mushrooms. This disease frequently occurs and there is no effective method for disease prevention and control. In the present study, *C. militaris* is found to be the only host of *C. cordycipiticola*, indicating strict host specificity. The infection process was monitored by fluorescent labeling and scanning and transmission electron microscopes. *C. cordycipiticola* can invade into the gaps among hyphae of the fruiting bodies of the host and fill them gradually. It can degrade the hyphae of the host by both direct contact and noncontact. The parasitism is initially biotrophic, and then necrotrophic as mycoparasitic interaction progresses. The approximate chromosome-level genome assembly of *C. cordycipiticola* yielded an N50 length of 5.45 Mbp and a total size of 34.51 Mbp, encoding 10,443 proteins. Phylogenomic analysis revealed that *C. cordycipiticola* is phylogenetically close to its specific host, *C. militaris.* A comparative genomic analysis showed that the number of CAZymes of *C. cordycipiticola* was much less than in other mycoparasites, which might be attributed to its host specificity. Secondary metabolite cluster analysis disclosed the great biosynthetic capabilities and potential mycotoxin production capability. This study provides insights into the potential pathogenesis and interaction between mycoparasite and its host.

## 1. Introduction

*Cordyceps militaris* (L.) Fr., one of the famous edible and medicinal fungi, has been widely used as an herbal drug and tonic in East Asia. The fruiting bodies have been cultivated artificially and it is estimated that the annual value of production of *C. militaris* is about 10 billion RMB in China [1]. During large-scale cultivation, fungal diseases frequently occur [2] and white mildew disease is one of the damaging diseases of *C. militaris* (Figure S1), resulting in a significant reduction in production and economic losses [3].

*Calcarisporium cordycipiticola* Jing Z. Sun, Cai H. Dong, Xing Z. Liu & K.D. Hyde, was firstly isolated from the infected fruiting body of *C*. *militaris* and belongs to the family Calcarisporiaceae of the order Hypocreales within the fungal phylum Ascomycota [4,5]. It was confirmed that *C. cordycipiticola* is the pathogen of white mildew disease of *C. militaris* by Koch’s postulates [3]. Some fungi that are consistently associated with other fungi are named as fungicolous (or mycophilic) fungi [6]. Biological characteristics of these fungicolous fungi have been reported before [3]. White colonies formed on the fruiting bodies of *C. militaris* (Figure S1) and a large number of conidia were produced in a short time, resulting in a rapid spread of the pathogen. There was no information on the infection process and pathogenic factors, which were crucial for disease prevention and control.

The interactions between fungicolous fungi and their hosts have been of considerable interest for a long time. Fungicolous fungi can infect their hosts *via* producing specialized structures such as appressorium and haustorium, and kill hosts through the secretion of enzymes or antibiotics, or by competition for nutrition and niches [7]. As for the fungal diseases of mushroom, some studies were performed on the pathogens of *Agaricus bisporus*, including wet bubble caused by *Hypomyces perniciosus* [syn: *Mycogone perniciosa*] [8], dry bubble by *Lecanicillium fungicola* [9], cobweb disease by *Cladobotryum dendroides* [10] and green mold by *Trichoderma aggressivum* [11]. *H. perniciosus* produced specific structures and invaded the hyphae of *A. bicospora* [12]. With electron microscopy and cytohistological techniques, Zhou found that *H. perniciosus* infected *A. bisporus* through the secretion of cell wall degradation enzymes and mechanical pressure from the appressorium [12]. *L. fungicola* did not produce a specialized structure, however, spores and hyphae of *L. fungicola* adhered to the surface of the host *A. bisporus,* and then protruded into the hyphae of the host [13]. Whether *C. cordycipiticola* can produce the specialized structure during infection, and how it interacted with the host *C. militaris* are our major concerns.

The parasitic lifestyle of some species of *Trichoderma* was related to the expansion of genes encoding fungal cell wall degrading enzymes and secondary metabolites (SMs) [14]. Comparative genomic and transcriptomic analyses revealed that there were significant expansions for protein families of transporters, cell wall degrading enzymes, cytochrome P450, and SMs in the genome of *H. perniciosus*, which were essential for the mycoparasitism of *A. bisporus* [8,15]. It was found that gene families with significant expansion in *C. dendroides* were mainly associated with the major facilitator superfamily (MFS) transporter, ankyrin repeats, and NACHT [NAIP (neuronal apoptosis inhibitory protein), CIITA (MHC class II transcription activator), HET-E (incompatibility locus protein from *Podospora anserina*) and TP1 (telomerase-associated protein)] domains, whereas P450 members were contracted noticeably [10]. Transcriptomic analysis of the interactions between *A. bisporus* and *L. fungicola* indicated that several oxidoreductases, cell wall degrading enzymes, ATP-binding cassettes (ABC) and MFS transporter proteins may play roles in the pathosystems [16]. Omics analysis will be helpful for the identification of pathogenicity-related genes and the characterization of pathogen-mushroom interactions.

Calcarisporiaceae and Cordycipitaceae are sister families in Hypocreales [5]. There is also much to be learned about the nature and evolution of interactions of *Calcarisporium* with host *Cordyceps* because of the relatively close phylogenetic relationship. Comparative genomic and phylogenomic analyses will identify the evolutional relationship, and genomic basis of their differing physiologies.

Mycotoxins are fungal SMs which have been associated with toxic effects to vertebrates, and mycotoxin contamination of food and feed is a threat to the health of humans and animals worldwide [17]. The safety of *C. militaris* has been confirmed since the genome did not contain genes for known mycotoxins [18]. Sometimes the fruiting bodies of *C. militaris* infected by *C. cordycipiticola* are mixed with normal *C. militaris* for sale, so whether *C. cordycipiticola* can produce mycotoxins is our concern. The genome data allows us to make a full view of *C. cordycipiticola* genes involved in biosynthesis of SMs for comparison with known mycotoxins such as citrinin, zearalenone, hypothemycin and solanapyrone.

In the present study, the genetic transformation and red fluorescent protein (RFP) labeling of *C. cordycipiticola* were developed. The infection process of *C. cordycipiticola* to the fruiting bodies of *C. militaris* was monitored by RFP-labeling, scanning electron microscopy (SEM) and transmission electron microscopy (TEM). The high-quality genome of *C. cordycipiticola* was sequenced by single molecule real-time (SMRT) and Illumina’s methods. Comparative genomic and SM cluster analyses were performed. It will be helpful for revealing the interaction between the pathogen and host, prevention and control of this disease.

## 2. Materials and Methods

### 2.1. Strains and Culture Conditions

*C. cordycipiticola* strain CGMCC 5.2193 was isolated from the diseased fruiting bodies of *C. militaris* strain CGMCC 3.16320 in this laboratory and inoculated on Potato Dextrose Agar (PDA) medium at 25 °C. *Escherichia coli* strain DH5α (Tiangen Biotech Co., Ltd., Beijing, China) was used for routine sub-cloning and cultured in Luria-Bertani medium at 37 °C. *Agrobacterium tumefaciens* strain AGL-1 (Tiangen Biotech Co., Ltd., Beijing, China) was used for the introduction of DNA into filamentous fungi and cultured at 28 °C in Yeast Extract and Beef medium supplemented with 50 μg/mL carbenicillin.

### 2.2. Agrobacterium tumefaciens-mediated Transformation (ATMT) of Calcarisporium cordycipiticola

For antibiotic sensitivity assays, conidia of *C. cordycipiticola* were spread on PDA plates with different concentrations of geneticin, glufosinate-ammonium, bleomycin and hygromycin B, respectively, and then incubated at 25 °C for 7 days. ATMT of *C. cordycipiticola* was performed following the method of species *Calcarisporium arbuscula* with some modifications [19]. The concentrations of acetosyringone (AS, 200, 400 and 600 μM), *A. tumefaciens* AGL-1 (OD_600_ 0.2, 0.4, 0.6 and 0.8), *C. cordycipiticola* conidia (10^5^, 10^6^ and 10^7^ conidia/mL), and days for co-culture (2, 3 and 4 days) were optimized.

### 2.3. Transformation of RFP-Tagged Calcarisporium cordycipiticola and Microscopy

Plasmid pFGL815-neoR-tubCp-RFP (kindly provided by Professor Xuming Mao, Institute of Pharmaceutical Biotechnology, Zhejiang University) containing resistant genes to kanamycin and geneticin, and RFP gene was transformed into *C. cordycipiticola*. Transformants expressing RFP were selected by PCR and fluorescent with ZEISS LSM700 laser scanning confocal microscopy (LSCM) (Zeiss, Jena, Germany).

The fruiting bodies of *C. militaris* strain CGMCC 3.16320 were cultivated according to our procedure [20]. Wild type of *C. cordycipiticola* and transformant expressing RFP were inoculated on the surface of fruiting bodies, respectively and the infection process was observed by LSCM after inoculation for 0, 3, 7, 15, 23 and 30 days. The red fluorescence was detected with an emission filter (excitation wavelength of 555 nm and emission wavelength of 580 nm). Photographs were taken with a Zeiss AxioCam MRc 5 digital camera (Göttingen, Germany) attached to an Imager A1 microscope (Göttingen, Germany).

Dual culture on PDA plates was performed between *C. cordycipiticola* labeled by RFP and *C. militaris*, which were inoculated at opposite sides of a 9-cm-diameter Petri plate. Sterile cover glasses were inserted and then incubated at 20 °C in darkness for 10 days. After two colonies contacted each other at about 24–48 h, the cover glasses were pulled out and observed by LSCM.

For observation by SEM, the samples (0.5 cm in length) after being infected for 3 d were processed following the method of Nunes et al. [13] with modifications. The samples were fixed in 2.5% glutaraldehyde in 0.05 M phosphate buffered saline (pH 7.2) for 8 h at 4 °C, and then washed with deionized water for 7 min. Samples were then washed again in cacodylate buffer for 8 min, dehydrated in graded ethanol and dried in a fume hood using critical point dryers (Autosamdri® 931, Tousimis, MD, USA) with CO_2_. Finally, samples were sputter-coated with gold by ion sputter coater (ISC150, SuPro Instruments, Shenzhen, China) under vacuum of lower than 1–2 Pa, a voltage of 110 V, frequency of 50/60 Hz, current of 10 mA and deposition time of 60 s and observed under SEM (SU8010, Hitachi, Tokyo, Japan). Secondary electron imaging mode was used with an accelerating voltage of 3–5 kV and effective working distance of 8 mm.

For observation by TEM, the samples (0.3 cm in length) being infected for 3 days were processed following the method of Ayache et al. [21] with modifications. The samples were fixed for 30 min in 2.5% glutaraldehyde and 1% osmium for 2 h. After washing three times for 10 min in 0.1 M phosphate buffered saline, samples were dehydrated in graded ethanol. Samples were then infiltrated with resin:70% ethanol (1:1) for 2 h at room temperature, in fresh pure resin for 1 h and then in fresh pure resin for overnight on an agitating device. Polymerization was carried out in an oven at 60 °C for 24 h. Sections thickness of 20–30 nm were cut transversely under ultrathin microtome (EM UC7, Leica, Weztlar, Germany). Finally, the samples were observed under TEM (HT7800, Hitachi, Tokyo, Japan) with an accelerating voltage of 80 kV and nominal resolution of 0.2 nm.

### 2.4. Infection of Calcarisporium cordycipiticola to the Other Species of Cordycipitaceae

The synnemata of some species of Cordycipitaceae, including *Beauveria bassiana* CGMCC 3.3575, *Cordyceps tenuipes* DCH268 and *Isaria cicadae* DCH463, were cultured following the methods of our previous report [22]. *C. cordycipiticola* strain CGMCC 5.2193 was cultured on PDA plates under dark at 25 °C for 7–10 days and discs of mycelia (0.5 cm in diameter) were cut with a sterile blade. Six pieces of mycelia were inoculated on the surface of synnemata in each cultivation bottle and cultured for 30 days.

### 2.5. DNA/RNA Preparation

The single conidium isolation CC01 from *C. cordycipiticola* strain CGMCC 5.2193 was cultivated in Potato Dextrose Broth at 25 °C and 150 rpm for 3 days. Mycelia were collected from the media by centrifugation at 5310× *g* for 20 min and ground in liquid nitrogen. Genomic DNA was extracted using a modified cetyltrimethylammonium bromide method [23]. For transcriptome analysis, *C. cordycipiticola* was grown on PDA medium at 25 °C for 7 days. Total RNA was extracted using E.Z.N.A.™ Plant Kit (Omega, Stamford, CT, USA).

### 2.6. Genome Sequencing and Assembly

The *C. cordycipiticola* genome was sequenced by Illumina and SMRT methods [24] by Nextomics Biosciences Co., Ltd. (Wuhan, China). DNA libraries with 400 bp and 20 kb inserts were constructed. The DNA sample of 400 bp library was quantified using NanoDrop 2000 (Thermo Fisher Scientific, Waltham, MA, USA), Qubit 3.0 (Invitrogen, Carlsbad, CA, USA), 2100 Bioanalyzer (Agilent, Santa Clara, CA, USA) and subjected to paired-ended 150 bp sequencing by Illumina HiSeq X10. The sequencing data (filtered reads: 5.50 Gbp, sequencing depth: 155×) were used to estimate the genome size, repeat content, and heterozygosity. The 20 kb library was quantified by the same ways, sequenced by SMRT using PacBic Sequel (Pacific Biosciences of California, Inc. Menlo Park, CA, USA), and the sequencing data (filtered reads: 5.93 Gbp, sequencing depth: 170×) were assembled by Canu v. 1.7 [25] with default parameters. Finally, Illumina reads were used for error correction and gap filling with SOAPdenovo GapCloser v. 1.12 [26]. The completeness of the assembled genome was evaluated using CEGMA [27] and BUSCO v. 3 [28]. The genome was deposited in the NCBI BioProject database https://www.ncbi.nlm.nih.gov/sra/PRJNA766243, accessed on 15 September 2021) under accession number PRJNA766243.

Transfer RNAs were predicted using tRNAscan-SE 2.0 [29], whereas rRNAs and noncoding RNAs were identified using RNAmmer 1.2 [30] and the the Rfam database [31].

RNA samples were sequenced by the Illumina method by Biomarker Technology Co., Ltd. (Beijing, China). Clean data were used for genome-assisted assembly. Quantification of gene expression levels was estimated by fragments per kilobase of per million mapped fragments (FPKM). The initial Illumina reads are available at the Sequence Read Archive of NCBI under accession number PRJNA766340.

### 2.7. Assembly and Annotation of Mitogenomes

Blast analysis was performed against *C. cordycipiticola* CC01 genome data using *C. militaris* mitogenome (KP719097) as the query. The mitogenome of *C. cordycipiticola* was first annotated automatically using the MFannot tool (http://megasun.bch.umontreal.ca/cgi-bin/mfannot/mfannotInterface.pl, accessed on 18 October 2020) based on genetic code, and then individually checked. Comparative analysis of mitochondrial genome was performed via online software Kalign [32].

### 2.8. Repetitive Sequences, Transposases, Repeat-Induced Point Mutation and Whole Genome DNA Methylation Analysis

Tandem repeats were identified by Tandem Repeats Finder v. 4.04 [33] by searching against the Repbase database [34]. Transposon elements (TEs) were excavated strictly using two software, including a de novo software RepeatModeler v. 1.0.11 (http://www.repeatmasker.org, accessed on 21 October 2020) and database-based software RepeatMasker v. 4.0.9 [35]. All the parameters were set as default. Repeat-induced point mutation (RIP) index was determined with the software RIPCAL by reference against the non-repetitive control families [36]. The transposons/retrotransposons encoding fragments were classified by blastp analysis against the Repbase. Whole-genome DNA modification detection and motif analysis were performed according to the method of Blow et al. [37] using the PacBio SMRT software v. 2.2.3 (http://www.pacb.com, accessed on 23 October 2020).

### 2.9. Genome Annotations

Protein-encoding genes were annotated by a combination of three independent ab initio methods, GeneMark v. 4.30 [38], SNAP [39], and GlimmerHMM [40]. Transcriptome data were incorporated into PASA (program to assemble spliced alignments) to improve the quality of *C. cordycipiticola* annotation [41]. Functional annotations for all predicted gene models were made using multiple databases, including TrEMBL, Swiss-Prot, nr, KEGG, GO, and KOG by blastp with an E-value of ≤1 × 10^−20^. Domain-calling analyses of protein-encoding genes were performed using the Pfam database [42] and HMMER [43].

### 2.10. Functional Annotation of Pathogenicity-Related Genes

The secretomes were identified based on recognizing the signal peptide and no transmembrane sequences. Proteins were considered to be secreted proteins if the signal peptides were identified by two methods, SignalP 5.0 [44] and TargetP 2.0 [45], and transmembrane sequences were not identified by at least one of the methods among SignalP 5.0, TMHMM 2.0 [46], and WoLF PSORT [47]. Effectors were predicted by EffectorP v. 2.0 [48]. Candidate pathogenic factors were predicted by sequence alignment against the pathogen-host interactions (PHI) database v. 3.5, by blastp with an E-value of <1 × 10^−20^ [49].

### 2.11. Phylogenomic Analysis

Phylogenomic analysis was performed using 32 fungal genomes with *Ustilago maydis* as an outgroup; 631 single-copy orthologs of the 32 fungi were identified using BUSCO v3 and were concatenated into one sequence using Sequence Matrix-Windows 1.7.8 [50]. The sequence alignment was done using MUSCLE v13 [51]. The PROTGAMMAILGF model was selected by ProtTest v. 3.4 [52]. A maximum likelihood (ML) phylogenomic tree was built using the 631 concatenated amino acid sequences with RAxML v. 8 [53]. Trees were figured in Tree v. 1.4.2 [54]. Bootstrap values higher than 50% from RAxML were indicated. The divergence time between species was estimated with the program r8s [55] using a Langley-Fitch model by calibration with the origin of the Ascomycota at 500 to 650 million years ago (MYA) [56].

### 2.12. Comparison Analysis of Carbohydrate-Active Enzymes

Carbohydrate-active enzymes (CAZymes) were identified using the dbCAN2 meta server (http://bcb.unl.edu/dbCAN2/, accessed on 30 October 2020) with HMMER [43]. Family-specific HMM profiles were downloaded from dbCAN CAZymes database [57]. The executable file hmmscan and the hmmscan-parser script (E-value ≤ 1 × 10^−17^ and coverage > 45%) provided by dbCAN were used to generate and extract the searching results. CAZymes of 13 other fungal species were analyzed with the same methods and compared with those of *C. cordycipiticola*.

### 2.13. Analysis of Genes Involved in Secondary Metabolism

SM clusters were predicted with fungal anti-SMASH 5.0 [58]. The core genes were annotated using stand-alone blast against Swiss-Prot and pfam databases. Domains were predicted using PKS/NRPS analysis website (http://nrps.igs.umaryland.edu/, accessed on 20 May 2021) and extracted manually. For phylogenetic analysis, the domain sequences were aligned by MUSCLE v13 and the tree was generated using a Dayhoff model with 1000 bootstrap replications, and pair-wise deletions for gaps or missing data. A neighbor-joining (NJ) tree was built by MEGA 7 [59] based on the ketoacyl CoA synthase (KS) amino acid sequences.

### 2.14. Experimental Design and Statistical Analysis

A flow chart of the whole experimental design was shown in Figure S2. For antibiotic sensitivity assays, optimization of ATMT and infection experiments, three technical replicates were used in a randomized design. The data were analyzed by one-way analysis of variance (ANOVA). The Tukey’s test was used for comparing more than two datasets, whereas the Student’s *t*-test was used for pairwise comparisons. The asterisks above the error bars represented means that were statistically different at: * *p* < 0.05; ** *p* < 0.01; *** *p* < 0.001. Data analyses were completed with SPSS 19.0 (SPSS, Inc., Chicago, IL, USA) and OriginPro 8.5 (OriginLab Corporation, Northampton, MA, USA). The graph was constructed using GraphPad Prism 8.0 (GraphPad Software, Inc., San Diego, CA, USA).

## 3. Results

### 3.1. Development of ATMT for Efficient Transformation of Calcarisporium cordycipiticola and RFP-Labeling

Antibiotic susceptibility testing showed that *C. cordycipiticola* was sensitive to geneticin and glufosinate-ammonium, and insensitive to bleomycin and hygromycin B (Figure S3). The high transformation efficiency of ATMT was observed with *C. cordycipiticola* conidia concentration of 1 × 10^7^ conidia/mL, co-cultured for 3 days, *A. tumefaciens* OD_600_ of 0.8 and AS concentration of 400 µM (Figure S4). Transformants expressing RFP were selected by PCR validation (Figure S5A) and strong red fluorescence was observed by LSCM (Figure S5B).

### 3.2. Infection Process of Calcarisporium cordycipiticola to the Fruiting Body of Cordyceps militaris

**Figure 1:**
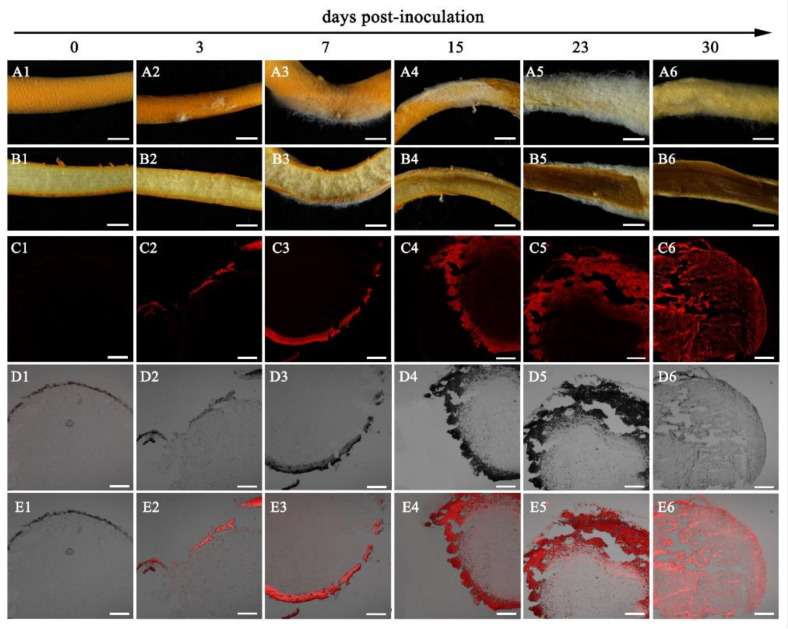
Infection process of *Calcarisporium cordycipiticola* to the fruiting bodies of *Cordyceps militaris* revealed by RFP labeling. (**A1**–**A6**), (**B1**–**B6**) and (**C1**–**C6**) the surface, longitudinal and cross sections of the fruiting bodies of *C. militaris* after being infected. (**C1**–**C6**), (**D1**–**D6**) and (**E1**–**E6**) observed by RFP labeling. (**C1**–**C6**) fluorescent micrograph of the infected samples. (**D1**–**D6**) brightfield micrograph of C1–C6. (**E1**–**E6**) overlay of fluorescent and brightfield micrographs. Scale bar = 2 mm in A1-B6 and 200 µm in C1–E6.

The visible mycelia of *C. cordycipiticola* on the fruiting bodies of *C. militaris* were observed at 3 days post-inoculation (dpi, Figure 1A1,A2) and the section of fruiting bodies of *C. militaris* showed as a pale yellow based on the handbook of Kornerup and Wanscher [60] (Figure 1B2), being consistent with the control (Figure 1B1). Then, the mycelia of *C. cordycipiticola* proliferated on the surface of fruiting bodies of *C. militaris* and covered them completely at about 20–30 dpi, and the color of the surface of the fruiting bodies showed as greyish-yellow at 30 dpi (Figure 1A3–A6). On the contrary, the color of the interior of the fruiting bodies became deeper gradually and showed as brown with liquid flowing out at last (Figure 1B3–B6). More and more intense red fluorescence was observed as the infection progressed (Figure 1C2–C6). The pathogenic hyphae can invade and then fill the fruiting bodies of *C. militaris* within 30 days (Figure 1C1–C6,E1–E6).

The dual culture of *C. militaris* and the RFP transformant of *C. cordycipiticola* on PDA plates confirmed that the hyphae of the two species were in a mixed arrangement, and there was no hyphal intertwinement, no haustorium and appressorium formation (Figure S6). Neither evident growth inhibition nor mycelia shrinking of *C. militaris* were observed even after being cultured for 10 days.

**Figure 2:**
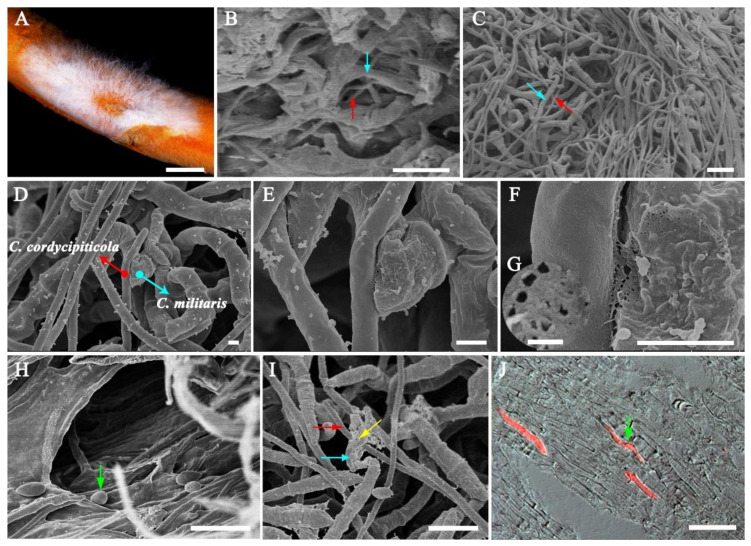
Interaction between *Cordyceps militaris* and *Calcarisporium cordycipiticola* observed by SEM and RFP labeling. (**A**) The fruiting body of *C. militaris* infected by *C. cordycipiticola* used for SEM observation. (**B**) The pathogen invaded into the gaps among the hyphae of *C. militaris* fruiting bodies. (**C**) Mixed arrangement of the two fungal hyphae. (**D**–**F**) The hyphae of *C. militaris* and *C. cordycipiticola* contacted with each other; (**E**–**G**) showed hyphal shrinking of *C. militaris* and some micropores. (**H**) The pathogen (hyphae and conidia) invaded into the fruiting bodies along the gaps. (**I**) A small number of pathogenic hyphae can penetrate into the host hyphae. (**J**) The conidia of *C. cordycipiticola* in the fruiting bodies of the host germinated (longitudinal section) observed by RFP labeling. Red and blue arrows indicated hyphae of *C. cordycipiticola* and *C. militaris,* respectively. Yellow arrow indicated that the pathogenic hyphae penetrated into the host hyphae and green indicated the conidia of *C. cordycipiticola.* Scale bar = 1 mm in A; 10 µm in B, C, H–J; 1 µm in D–F and 100 nm in G.

**Figure 3:**
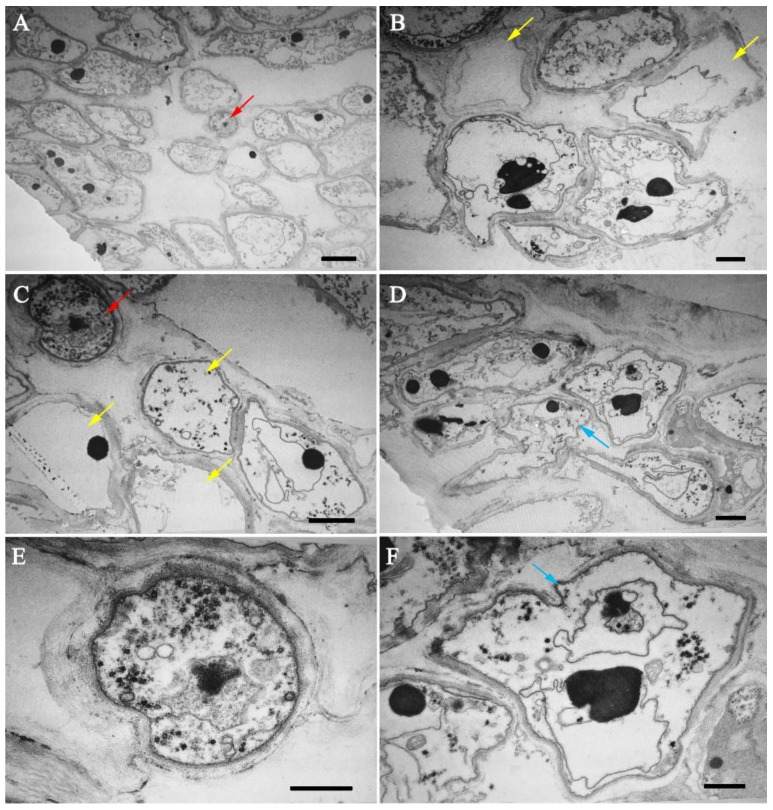
Cell morphologies of *Cordyceps militaris* and *Calcarisporium cordycipiticola* during infection observed by TEM. Cross sections of the infected fruiting body of *C. militaris* (3 dpi) were shown. (**A**), (**C**) Hyphal cells of *C. cordycipiticola* and *C. militaris*. (**E**) Hyphal cell of *C. cordycipiticola*. (**B**–**D**,**F**) Hyphal cells of *C. militaris* after being infected by *C. cordycipiticola*. Red arrow indicated cells of *C. cordycipiticola*, yellow indicated empty hyphal cells of *C. militaris* without organelles, and blue indicated the deformed cells. Scale bar = 1 µm.

The samples at 3 dpi (Figure 2A) were observed by SEM and TEM. It is easy to distinguish the hyphae of these two fungi under SEM since the diameter of *C. militaris* hyphae is 2.02 ± 0.39 µm, almost twice of *C. cordycipiticola* (1.01 ± 0.20 µm, Figure 2C,D,I). The two kinds of fungal hyphae were in mixed arrangement (Figure 2C), however, there was no intertwinement of the hyphae as observed in some species of *Trichoderma* [61]. There were some gaps among hyphae of the fruiting body of *C. militaris* [62] and the pathogen invaded into these gaps until being full of the entire fruiting bodies (Figure 2B,H). It was observed that some hyphae of these two fungi can stick together (Figure 2D–F), with some sticky substance at the touch point (Figure 2E,F), where some micropores were produced (Figure 2F,G) with hyphal shrinking (Figure 2E,F). Some hyphae of *C. militaris* which were not in touch with *C. cordycipiticola* directly also became shrinking (Figure 2D,E). The haustorium and appressorium were not observed during the infection (Figure 2D–F and Figure 3). A small number of pathogenic hyphae can penetrate into the host hyphae (Figure 2I). Conidia entered into the fruiting body along the gaps and germinated, accelerating the spread of disease (Figure 2H–J).

Under TEM, the cell walls of the pathogen were intact (Figure 3A,C,E) whereas cells of *C. militaris* were deformed whether they were in touch or not touch with the hyphae of the pathogen directly (Figure 3A,C,D,F). The organelles and cytoplasm of the hyphal cells of *C. militaris* were dissolved, resulting in hollow hyphal cells even if the hyphae had not burst (Figure 3B,C). Finally, the cells were broken and died, leading to the wilting of fruiting bodies.

### 3.3. Host Specificity of Calcarisporium cordycipiticola

**Figure 4:**
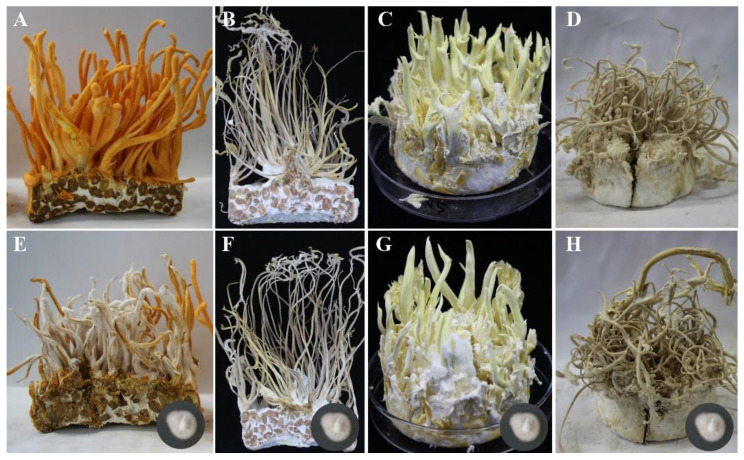
Infection on different species of Cordycipitaceae. (**A**–**D**) were *C. militaris*, *C. tenuipes*, *B. bassiana*, and *I. cicadae* that were cultivated on the wheat media, respectively. (**E**–**H**) are those infected by *C. cordycipiticola* for 30 days. Three biological replicates for each species.

After inoculation on the synnemata of *B. bassiana, C. tenuipes*, and *I. cicadae* for 30 days (Figure 4A–H), the mycelia of *C. cordycipiticola* cannot proliferate and the inoculation site remained unchanged. There was no pathogenic sign (Figure 4F–H). However, the surface of the fruiting bodies of *C. militaris* has been fully covered by the mycelia of *C. cordycipiticola* (Figure 4E). It seemed that the pathogen *C. cordycipiticola* can only infect *C. militaris.*

### 3.4. General Features of the Calcarisporium cordycipiticola Genome

In order to investigate the infection mechanism, the genome of *C. cordycipiticola* was sequenced by both Illumina and PacBio SMRT technologies. Subreads distribution analyses confirmed the high quality of the 20 kb library (Figure S7). The resulting assembly yielded an N50 length of 5.45 Mbp and a total size around 34.51 Mbp (Table 1), which was similar to *Calcarisporium* sp. 525 (36.8 Mbp) [63] and *C. militaris* (32.2 Mbp) [18], less than that of *C*. *arbuscula* (45.0 Mbp) [64].

**Figure 5:**
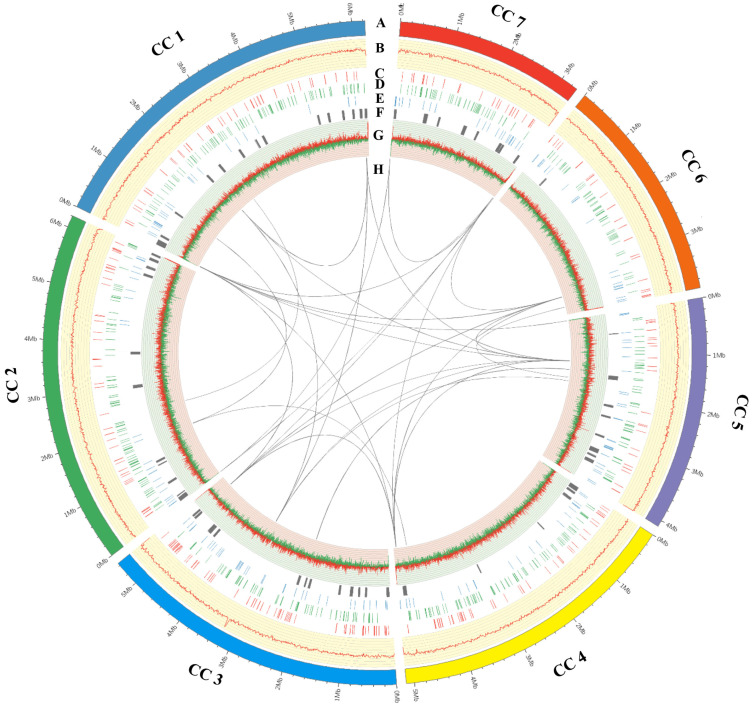
Circos-plot of genome of *Calcarisporium cordycipiticola*. The Seven scaffolds of *C. cordycipiticola* were displayed by circos-plot (Mbp scale). The circos from outside to inside were: (A) Seven chromosomes almost; (B) DNA methylations; (C) Carbohydrate-active enzymes; (D) PHI-base genes; (E) Putative effectors; (F) Clusters of secondary metabolites; (G) GC content; (H) Duplicated genes. DNA methylations and GC contents were statistical results of 10 kb non-overlapping windows. The inner lines link duplicated genes.

The sequenced data were assembled into eight scaffolds, an approximate chromosome-level, with three of these scaffolds containing characteristic telomeric (CCCTAA)n or (TTAGGG)n repeats on both the 5′ and 3′ ends of the sequence, and four having telomeric repeats on one of the 5′ or 3′ ends of the sequence. The three scaffolds with telomeres on both ends were taken to be fully sequenced chromosomes. The remaining one scaffold was evaluated as mitochondrion genes and fragments that were not integrated into the genome (Table S1). The seven assembled scaffolds which ranged in size from 3.3 to 6.2 Mbp were displayed by circos-plot (Figure 5, Table S1). The related species *C. militaris* contained seven chromosomes ranging in size from 1.9 to 8.3 Mbp [65]. The mitogenome of *C. cordycipiticola* is 25,762 bp (Figure S8), containing 15 common protein-coding genes, two rRNA and 24 tRNA genes, which was highly similar to that of *C. militaris* (Table S2).

A total of 10,443 protein-coding genes with an average sequence length of 1,692.74 bp were predicted, in which the gene density was 302 gene per 1 Mbp. Three-hundred-and fifty-six tRNA genes and 467 pseudogenes were predicted in the genome which was much more than that of *C. militaris* (Table 1). The length of protein-coding genes was 17.7 Mbp (almost 50%, 17.7 /34.51 Mbp) and each protein-coding gene had about 2.84 exons and 1.84 introns. 99.3% (9612) putative protein-coding genes were supported by RNA-seq data. Overall, 10,413 (99.71%) of the predicted proteins had known homologues in at least one functional protein database (Figure S9, Table S3). The completeness of genome assembly and annotation with single-copy orthologs test suggested a well-completed annotation set, with 94.7% of the Fungi BUSCOs being present within the RefSeq annotation set (Figure S9).

According to the GO database, 4944 annotated genes were assigned to GO categories, with the first three as “metabolic process”, “catalytic activity” and “cellular process” (Table S4). By mapping to the KEGG database, “global and overview maps” accounted for the maximum proportion of KEGG annotations. The first three were biosynthesis of amino acids, biosynthesis of antibiotics and carbon metabolism (Figure S10, Table S5).

**Figure 6:**
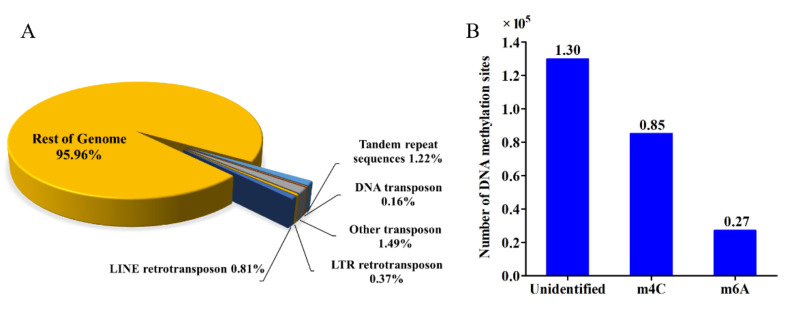
Repeat elements and DNA methylation sites of *Calcarisporium cordycipiticola*. (**A**) The percentage of different types of repetitive sequences in the *C. cordycipiticola* genome. (**B**) Statistic analysis of candidate DNA methylation sites from primary sequence of *C. cordycipiticola* genome.

Approximately 4.04% of the repeat sequences that included TEs (~2.82%) and tandem repeats (~1.22%) were identified in *C. cordycipiticola* genome. The Class I (retrotransposons) and Class II (DNA transposons) TEs occupied 1.18% and 0.16% of the genome (Figure 6A). Class I (retrotransposons) mainly included *Gypsy* and *Tad1* and Class II (DNA transposons) mainly included *MULE-MuDR* and *hAT-Charilie* (Figure S11A). *C. cordycipiticola* exhibited a little expansion of repeat content compared to some other ascomycetes (Table S6). The repeat sequence of congeneric fungi, *Calcarisporium* sp. KF525 and *C. arbuscula* NRRL 3705, only accounted for 1.28% and 3.08% of their genome sizes, respectively [63,64]. It was reported that two mycoparasite species, *Trichoderm atroviride* and *T. virens*, only had 0.49% and 0.48% repeat sequences [14].

RIP is a genome defense mechanism in fungi during which duplicated sequences are mutated from CpA to TpA [66]. Sequences that have been subjected to RIP were expected to have a high TA/AT ratio and low (CA + TG)/(AC + GT) ratio, with values >0.89 and <1.03, respectively [36]. A significant RIP was observed in *C. cordycipiticola* genome by the high value of TpA/ApT (2.03) and the low ratio of (CpA + TpG)/(ApC + GpT) (0.64) (Figure S11B). One homologue (CCOR_04202) of RIP defective-1 of *Neurospora crassa* (NCU02034) required for RIP was identified in *C. cordycipiticola* genome (identity of 39% and E-value of 3 × 10^−90^). Transcriptome analysis showed that this gene expressed with FPKM of 0.27, 0.75 and 0.37 for the three repeats in the mycelial sample and there was no increase during the infection process. It was indicated that there was indeed RIP defense mechanism in *C. cordycipiticola* which was not related to the infection.

DNA methylation is involved in many important cell processes, such as genomic imprinting and gene transcription regulation [67]. Using SMRT, 6-methyl-adenosine (m6A) and 4-methyl-cytosine (m4C) methylation were detected in particular. In total, 85,229 m4C and 27,110 m6A were identified in the *C. cordycipiticola* genome. However, the majority of methylation sites (129,857) were uncategorized (Figure 6B). Interestingly, most of the categorized DNA methylations are m4C, accounting for 75.9%, whereas m6A only accounts for 24.1%.

### 3.5. Phylogenomic and Evolution Analyses

A ML phylogenomic tree was generated based on 631 single-copy orthologs of 32 fungi, including insect pathogens, plant pathogens, nematode parasites, mycoparasites and saprophytes, with *U. maydis* as the outgroup (Table S7). The results verified that *C. cordycipiticola* was a distinct lineage (Calcarisporiaceae) from other families in Hypocreales, as described by Sun et al. [5].

**Figure 7:**
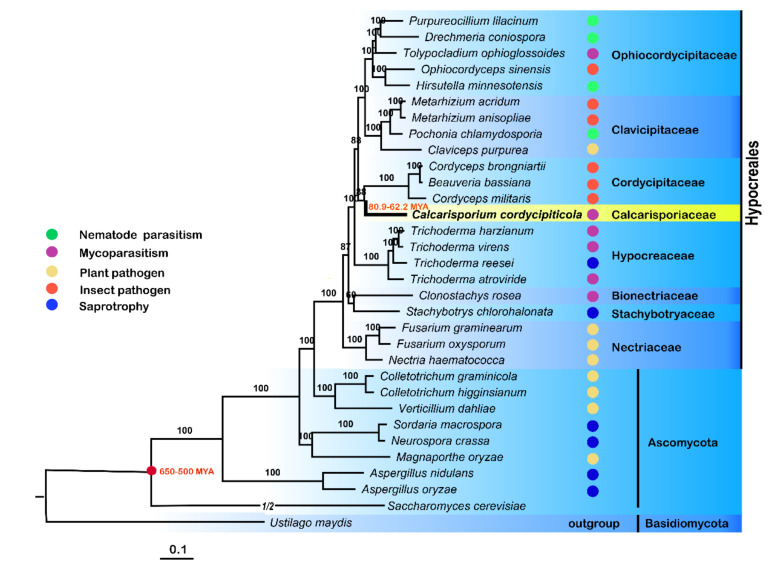
Phylogeny of *Calcarisporium cordycipiticola* and other 30 sequenced Ascomycota genomes. A ML phylogenomic tree showed the evolutionary relationship of *C. cordycipiticola* with different fungal species. Bootstrap values were shown beside the nodes. Life strategies and host preferences were indicated.

The inferred phylogeny illustrated that *C. cordycipiticola* was evolutionarily close to its host *C. militaris*, and they diverged after a split with *Trichoderm* spp., which was consistent with the report that *B. bassiana* and *C. militaris* diverged after a split with *Trichoderma* spp. [68]. *C. cordycipiticola* diverged before *C. militaris* (Figure 7).

The branch of *B. bassiana*, *Cordyceps* *brongniartii* and *C. militaris* diverged from *C. cordycipiticola* around 80.9–62.2 MYA (Figure 7) after or at the cretaceous extinction event (65 MYA) [69]. This phylogeny also reinforced the previous result that the split between Cordycipitaceae and Clavicipitaceae occurred before Ophiocordycipitaceae diverged from Clavicipitaceae (Figure 7) [70].

The earliest diverging group in Nectriaceae of Hypocreales comprised primarily plant pathogenic species, including *Fusarium graminearum*, *Fusarium oxysporum* and *Nectria haematococca* (Figure 7). Comparative genomic studies supported that the ancestral state of *Hypocrea*/*Trichoderma* was mycoparasitic [14]. The four nematode parasitic fungi, *Drechmeria coniospora*, *Hirsutella minnesotensis*, *Pochonia chlamydosporia* and *Purpureocillium lilacinum*, clustered with insect pathogens, indicating that nematode and insect pathogens might share a common ancestor. The host analysis supported a major transition within the order from plant hosts/substrates in early diverging lineages to fungal hosts and then to insect and nematode hosts.

### 3.6. The Secretome and Potential Eeffectors

Secreted proteins are important for the pathogenicity of parasitic fungi. A total of 942 sequences were identified to encode proteins with signal peptides but not transmembrane helices (Table S8). The proportion of genes encoding secreted proteins (9.02%) was similar to 7–10% in plant pathogens [71]. KEGG pathway enrichment analysis indicated that the putative secreted proteins were mainly involved in primary metabolism, especially sugar metabolism, including starch and sucrose metabolism (ko00500), amino sugar and nucleotide sugar metabolism (ko00520), glycosaminoglycan degradation (ko00531), galactose metabolism (ko00052), other glycan degradation (ko00511) and N-glycan biosynthesis (ko00510) (Figure S12A).

In the fungal plant pathogens, pathogen-secreted effectors play critical roles in facilitating the proliferation of pathogens, often by suppressing the host immune system [72]. These secretomes were further examined for effector candidates and 189 proteins were obtained (Table S8) based on the assumption that fungal effectors are small cysteine-rich proteins with fewer than 400 amino acids and more than four cysteine residues [73].

There were 434 putative pathogenic factors in *C. cordycipiticola* annotated by the PHI database (Table S8). KEGG pathway analysis indicated that they were mainly enriched with MAPK signaling pathway (ko04011), AGE-RAGE signaling pathway in diabetic complications (ko04933) and ABC transporters (ko02010) (*p* < 0.001, Figure S12B).

### 3.7. Carbohydrate-active Enzymes (CAZymes)

CAZymes which can degrade and modify polysaccharides might be required when *C. cordycipiticola* degraded the structural polysaccharide armor of the host, such as chitin, during the course of its parasitism. A detailed examination of the CAZymes of *C. cordycipiticola* was performed and compared with other fungi, including mycoparasitic fungi (*Clonostachys rosea*, *Trichoderma atroviride* and *Trichoderma harziam*), nematode parasitic fungi (*H. minnesotensis* and *P. chlamydosporia*), entomopathogenic fungi (*B. bassiana*, *C. militaris* and *Ophiocordyceps sinensis*) and plant pathogenic fungi (*Botrytis cinerea*, *F. graminearum*, *Magnaporthe oryzae* and *Verticillium dahliae*) (Table 2). Four hundred and forty-one CAZymes were identified in the genome of *C. cordycipiticola* (Table 2), which was much less than the other mycoparasitic fungi, *C. rosea* (656), *T. harziam* (516) and *T. atroviride* (473). In general, the number of CAZymes of mycoparasites (average 522) was a little less than that of plant pathogenic fungi (average 561) but was much more than that of entomopathogens (average 307, *p* = 0.019).

There were also significant differences in the spectrum of CAZymes produced by mycoparasitic fungi. Compared to mycoparasitic fungi, *C. rosea*, *T. atroviride* and *T. harzianum*, CAZymes candidates of *C. cordycipiticola* were radically lower in number for each class except AAs (Auxiliary activity family) (Table 2).

Seventy-seven families containing 211 genes in *C. cordycipiticola* that encoded glycoside hydrolases (GHs) were identified (Table S9). The number of GHs of mycoparasitic fungi (average 250) was close to the plant pathogens (average 246), and much more than that of entomopathogenic fungi (average 134, *p* = 0.012), indicating that GHs were more important for mycoparasitic and plant pathogenic fungi. The most abundant family for *C. cordycipiticola* was GH16 (β-glucanases) and GH18 (chitinase) (Table S9), both had 16 encoding genes that might be responsible for the cell wall degradation of the host. Family GH18, enzymes involved in chitin degradation, was also strongly expanded in *Trichoderma* [74]. Among the 16 GH18 genes, 11 encoded secreted protein (Table S10).

Especially, the most abundant family of CBM (carbohydrate-binding module) was CBM18s, which was related to the high number of GH18 in this species (Table S11). However, it was reported there was a low number of GH18 and CBM18s in the mycoparasitic fungi *C. rosea* (Tables S9 and S11). The significant contraction of CBM1 (only 4) in *C. cordycipiticola* was observed compared to the mycoparasitic (average 18) and plant pathogenic fungi (average 21), and even lower than the average (12) of all the fungi tested. *C. cordycipiticola* also possessed several families of cellulases (GH6, GH7, GH12 and GH45) and other enzymes involved in degrading cell walls (GH11, GH30, GH51, GH53, GH62, GH67 and GH115) (Table S9).

Another major class of CAZymes, glycosyltransferases (GTs) which were involved in the biosynthesis of oligo and polysaccharides, were represented in the genome with 71 members in 34 families (Table S12). These enzymes exhibited less variability in ascomycetes. A series of carbohydrate esterase (CE)-encoding genes were also detected in the genomes, including the most abundant sterol esterases (CE10) and cutinases (CE5), which are virulence factors of some plant pathogens [68]. There was only 1 pectate lyase (9 to 25 in plant pathogens).

### 3.8. Great Biosynthetic Capabilities of Secondary Metabolites and Potential of Mycotoxin Biosynthesis in Calcarisporium cordycipiticola

Sixty-six SMs were identified in the genome of *C. cordycipiticola* (Table 3), similar to 60 SMs in marine fungus *Calcarisporium* sp. KF525 [63] and 65 in mushroom-endophytic fungus *C. arbuscula* NRRL 3705 [64]. However, compared to the sequenced species in Cordyciptaceae, the number of SMs in *C. cordycipiticola* (66) was much more than 30 SMs in *C. militaris* [18], 45 in *B. bassiana* [68]. It was indicated that the fungi of *Calcarisporium* genus had more biosynthetic gene clusters and had a large potential for SM production (Table 3).

The average number of core genes related to SM synthesis in ascomycetes is 48 [75]. However, 82 core genes were identified, including 20 type I polyketide synthase (T1PKSs), 17 non-ribosomal peptide synthases (NRPSs), 11 PKS-NRPS hybrids, 2 NRPS-PKS hybrids, 12 putative NRPS-like enzymes, four terpene, and 16 others as described in Table S13.

Forty-four SM clusters have no homologues blasting against the MiBiG database. Three PKS-NRPS clusters (cluster 8, 40 and 50) were predicted for pyranonigrin E, botryenalol and radicicol, respectively; two NRPS clusters (cluster 6 and 15) for dimethylcoprogen and aureobasidin A1; one T1pks cluster (cluster 65) for phomopsins, and one terpene cluster (cluster 49) for clavaric acid with 100% of similarity; one T1pks cluster (cluster 46) for depudecin with 50% of similarity (Table S13).

**Figure 8:**
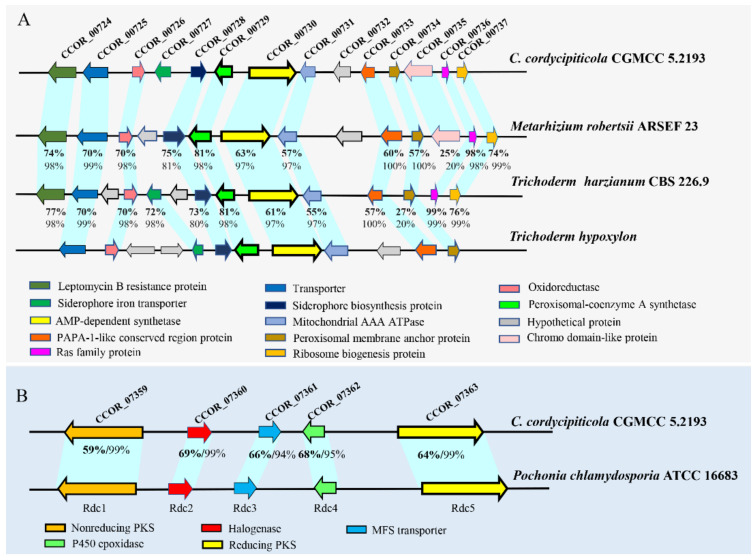
The putative biosynthetic gene clusters for coprogen and radicicol in the genome of *Calcarisporium cordycipiticola* CGMCC 5.2193. (**A**) Comparison with the coprogen cluster reported in *Metarhizium robertsii* ARSEF 23, *T. harzianum* CBS 226.9 and *T. hypoxylon*. (**B**) Comparison with the radicicol cluster reported in *P. chlamydosporia*. The identity and query cover of each homologue to *C. cordycipiticola* CGMCC 5.2193 counterparts were shown.

Blast analysis showed that CCOR_00730 of cluster 6 shared high similarity with *Trichoderm hypoxylon dfcA*, which was confirmed to be responsible for biosynthesis of coprogens and required for the inhibition of the pathogenic fungi *F. oxysporum* and *Mucor corcinelloides* [76]. The flanking genes, including the putative AMP-dependent synthetase /ligase, acetyltransferase, and siderophore transporter, also shared the high similarity with the biosynthetic gene cluster of amphiphilic coprogens in *T. hypoxylon* (Figure 8A). This cluster was widespread in fungi, and it was assumed that cluster of CCOR_00730 was responsible for the biosynthesis of coprogens in *C. cordycipiticola* (Table S14).

Five proteins encoding by genes of cluster 50 shared high sequence identity of 59–69% with the counterparts of Rdc1, Rdc2, Rdc3, Rdc4 and Rdc5 of *P. chlamydosporia* ATCC 16,683 (Figure 8B) which were confirmed to be responsible for radicicol biosynthesis [77]. Radicicol and analogs have significant antibacterial, antifungal, antiviral, anticancer and antiparasitic activities, and are also potent heat-shock protein 90 inhibitors [78,79]. Transcriptome analysis showed that all the genes of this cluster were expressed, though most were at low levels (Table S15).

Six proteins encoded by the genes of cluster 8 showed high sequence identity (31–52%) with a cluster of *Aspergillus niger* CBS 513.88 being responsible for pyranonigrins (Figure S13A) [80]. Pyranonigrins are a family of antioxidative compounds that are reported to be produced by *A. niger*, all featuring a unique pyrano [2,3-c] pyrrole core structure which is rarely found in nature [81]. Transcriptome data showed that these genes were moderately expressed in the mycelia (Table S15), indicating *C. cordycipiticola* might produce pyranonigrins-related compounds.

Depudecin is an inhibitor of histone deacetylase, similar to HC toxin. CCOR_06814 of cluster 46 showed the high sequence identity with DEP5 (identity of 54% and E-value of 0) which was confirmed to be involved in depudecin biosynthesis in *Alternaria brassicicola* ATCC 96,836 [82]. Moreover, the two FAD-dependent monooxygenase of cluster 46 showed sequence identity with DEP2 (identity of 50% and E-value of 0) and DEP4 (identity of 59% and E-value of 0) of *A. brassicicola* ATCC 96,836 (Figure S13B). However, transcriptome data showed CCOR_06808-CCOR_06814 were barely expressed in the mycelia (Table S15).

**Figure 9:**
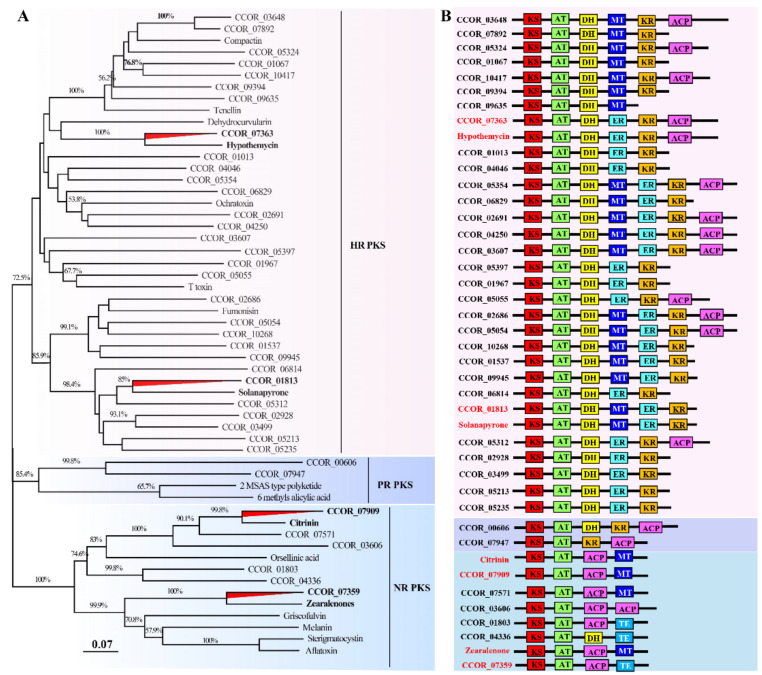
Phylogenetic and modular analyses of *Calcarisporium cordycipiticola* polyketide synthases compared with those involved in the production of mycotoxins. (**A**) A NJ tree showing the relationships of KS domain sequences. HR PKS, highly reducing PKS; PR PKS, partially reduced PKS; NR PKS, non-reduced PKS. (**B**) Modulation and comparison of *C. cordycipiticola* PKSs with those involved in production of mycotoxins. ACP, acyl carrier protein domain; AT, acyltransferase domain; DH, dehydratase domain; ER, enoyl reductase domain; KR, ketoreductase domain; MT, methyltransferase domain; TE, thioesterase domain. The different mycotoxin encoding PKSs used were listed in Table S17.

Phylogenetic analysis based on the 55 KS domain sequences of PKSs of *C. cordycipiticola* (Table S16) and 17 known mycotoxins (Table S17) indicated the groupings of some PKS proteins of *C. cordycipiticola* with those involved in mycotoxin-producing enzymes (Figure 9A). Modular analysis indicated that CCOR_07363 groups with hypothemycin-producing PKS, CCOR_01813 with solanapyrone, CCOR_07909 with citrinin and CCOR_07359 with zearalenone (Figure 9A) have similar domain organizations with the corresponding mycotoxin producing PKSs except for CCOR_07359 (Figure 9B).

Further survey showed that CCOR_01813 protein has 41% identity (E value of 0 and query cover of 99%) with SOL1 which has been confirmed for solanapyrone biosynthesis in *Alternaria solani* [83]. The cluster for solanapyrone included six genes (*sol1*, *sol2*, *sol3*, *sol4*, *sol5*, *sol6*), and homologous comparison found that the six proteins of the cluster 17 of *C. cordycipiticola* shared a sequence identity of 25–52% with those of *A. solani* (Figure S13C, Table S14). Transcriptome analysis showed that these genes were highly or moderately expressed in the mycelia (Table S15), indicating *C. cordycipiticola* might produce solanapyrone-related compounds.

For the cluster of CCOR_07363, it was found that core genes CCOR_07359 and CCOR_07363 shared the identity of 55% and 61% with HPM3 and HPM8 of *Hypomyces subiculosus* which were responsible for hypothemycin biosynthesis (Table S14) [84]. Besides, CCOR_07359 and CCOR_07363 proteins shared 54% and 59% identity with PKS13 and PKS4 for zearalenone biosynthesis in *F. graminearum* (Table S14). However, the flanking proteins for both hypothemycin and zearalenone biosynthesis clusters were not found in *C. cordycipiticola.*

CCOR_07909 protein had the identity of 52% (E value of 0 and query cover of 99%) with CitS responsible for citrinin biosynthesis in *Monascus ruber* [85]. Flanking proteins of the cluster, CCOR_07908 and CCOR_07910, showed high sequence identity with serine hydrolase (CitA) and oxidoreductase (CitC) of *M. ruber* (identity 60% and 50%) (Table S14). Transcriptome analysis showed that these three genes were highly expressed in the mycelia (Table S15), indicating *C. cordycipiticola* might produce citrinin-related compounds.

## 4. Discussion

Mycoparasitism is a lifestyle where a living fungus (host or prey) is parasitized by and acts as a nutrient source of another fungus (mycoparasite or predator) [86], which can cause devastating diseases of mushrooms in nature and industry by reducing the yield and quality worldwide if the host is mushroom [6]. In the present study, *C. cordycipiticola* was found to parasitize and degrade the hyphae of the fruiting body of *C. militaris*, by direct contact and noncontact (Figure 2). The approximate chromosome-level genome was assembled and some characteristics being consistent with mycoparasitic lifestyle were revealed. We showed specific mycoparasitism between two phylogenetically close species here.

*C. cordycipiticola* cannot infect the other species of Cordycipitaceae, including *B. bassiana, C. tenuipes* and *I. cicadae* (Figure 4)*,* indicating the specificity of the host. Until now, *C. militaris* is known to be the only host of *C. cordycipiticola.* This strict host specificity is not common. Species of the genus *Trichoderma* together with *C. rosea*, which are the most studied fungal mycoparasites, have wide host ranges comprising several plant pathogens [86]. For the congeneric fungi, *Calcarisporium parasiticum* has been observed to parasitize several species of *Physalospora* and closely related fungi [87]. *Scleromitrula shiraiana* is a causal agent of mulberry sclerotial disease with a narrow host range but includes some species of genus Morus [88].

The fruiting body of *C. militaris* was consists of prosenchyma and pseudoparenchyma tissues [62]. The prosenchyma tissues are loosely woven and have distinguishable and typical elongated hyphal cells lying parallel to one another [89]. There were some gaps among the hyphae of the fruiting body as shown in Figure 2 and Figure 3, which was consistent with the observation of Feng et al. [62]. *C. cordycipiticola* can invade into these gaps, filling the entire fruiting bodies gradually (Figure 1 and Figure 2). It was reported that plant stomata are not only essential for gas exchange with the environment and controlling water loss, but also as a checkpoint of host immunity and pathogen virulence [90]. Here it was suggested that the gaps among the hyphae of the fruiting body of *C. militaris* may play similar roles with the plant stomata, *viz* promote the respiration of hyphae inside fruiting bodies and become the channels for *C. cordycipiticola* to invade.

Some mycoparasites form specialized infection structures such as hook-like, braid-like, and clamp-like contact structures at host-parasite interfaces [91]. Invasive mycoparasites, such as *Trichoderma* species [92] and *C. rosea* [7] can form appressoria-like infection structures aiding in penetration. *C. parasiticum* can form a small ’buffer’ cell at the tip of a hypha where it contacts the host [87,93]. In the present study, we have observed the whole infection process carefully, focusing on the specialized infection structures, however, we cannot find any (Figure 2 and Figure 3). It was only observed that the hyphae of these two fungi were closely appressed, and then produced some micropores at the interface (Figure 2F,G). Finally, the hyphal cells of the host were deformed and the protoplasm was discharged, resulting in the empty cells (Figure 3B–D). During the infection, only a few pathogenic hyphae were observed to penetrate into the host hyphae (Figure 2I).

**Figure 10:**
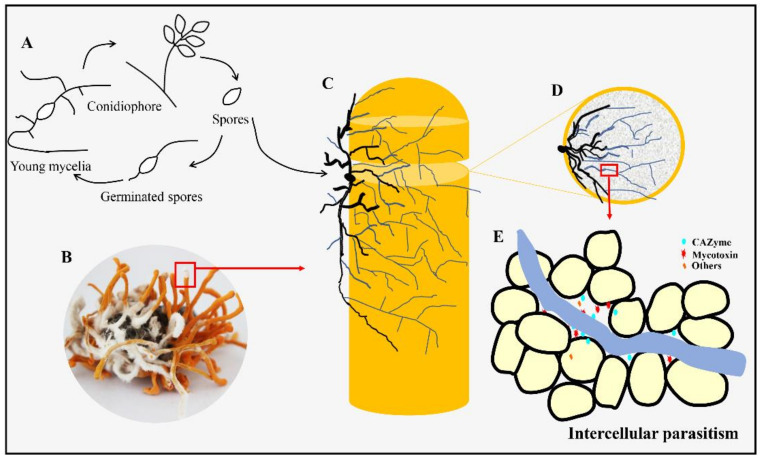
Model of pathogenic process of *Calcarisporium cordycipiticola*. (**A**) The asexual life cycle of *C. cordycipiticola*. (**B**) Fruiting body infected by *C. cordycipiticola*. (**C**–**E**) Model of colonization and invasion.

Mycoparasitism can be largely categorized into two groups, biotrophic and necrotrophic parasitisms according to the trophic mechanism [86]. Necrotrophic mycoparasites tend to have a broad host range, with relatively unspecialized parasitic mechanisms, and kill their host. In contrast, the biotrophic relationships between one fungus and another are complex, controlled and relatively non-destructive, and often, but not always, with narrow host ranges that have co-evolved [94]. Based on the strict host specificity, the observation that micropores formed between the appressed hyphae of host and parasite, as well as a few hyphae that penetrated into the host hyphae and that host cells remained functional at the early stage of infection, it was proposed that *C. cordycipiticola* was firstly biotrophic. However, it turned to necrotrophy as the mycoparasitic interaction progressed. *C. cordycipiticola* infected the fruiting bodies of *C. militaris* (Figure 10) and shuttled among the host hyphae (Figure 10E). The cell of *C. militaris* was deformed with hyphal shrinking till plasmatorrhexis. *C. cordycipiticola* derived nutrients from the disintegrated host, resulting in the wilting of the fruiting body of *C. militaris*. In a word, *C. cordycipiticola* is a destructive mycoparasite of *C. militaris*.

The pathogen *C. cordycipiticola* is an invasive type, similar to deep fungal infections of animal [95], which can well explain the destructiveness of the disease. Once the symptoms were visible to the naked eye, the pathogen had already entered the fruiting bodies. Quick germination of a large number of conidia, the rapid expansion of hyphae [3] and intruding into the fruiting bodies resulted in the spread of disease in a short time.

*C. cordycipiticola* can be cultured on artificial media besides mycoparasitism, but no morphologic alteration of *C. militaris* was observed when the dual culture of *C. militaris* and *C. cordycipiticola* on PDA plates was performed (Figure S6). The same results were observed with dual culture between *A. bisporus* and *L. fungicola* [96], *A. bisporus* and *H. perniciosus* [97]. It was suggested that the interactions between mushrooms and pathogens on plates (pythogenesis) are different from infecting on the fruiting bodies (mycoparasites). *C. cordycipiticola* may initiate different lifestyles when facing different living environments, which has also been reported in some mycoparasites such as *Saccharomycopsis schoenii* [98].

Sequencing and analyses of the genomes of mycoparasites *Trichoderma* spp. [14] and *C. rosea* [7], as well as *Tolypocladium ophioglossoides* [99] and *Escovopsis weberi* [100] in the order Hypocreales, revealed several gene families with a mycoparasitic-related expansion, but distinct differences also emerged among the different mycoparasites. The genome size, structure, and gene content are influenced heavily by natural selection and governed by the lifestyle and ecological niche of a species [86]. The *C. cordycipiticola* genome size of 34.51 Mbp is similar to genomes of mycoparasites *Trichoderma* spp. (vary between 31.7 and 39.0 Mbp) and the truffle parasite *T. ophioglossoides* (31.2 Mbp), but a pronounced reduction than that of *C. rosea* (55.18 to 70.7 Mbp) [7,101]. This reduction in genome size is accompanied by a lower number of protein-encoding genes (>14,268 in *C. rosea* and 10,443 in *C. cordycipiticola*).

Fungal secreted proteins, effectors, cell wall degrading enzymes and SM genes are known to be under positive selection in pathogenic fungi [102]. There were some characteristics for the genome of *C. cordycipiticola* which were consistent with the mycoparasitic lifestyle. The proportion of genes encoding secreted proteins (9.02%) is relatively high; the pathogen-secreted effectors were mainly enriched with amino sugar and nucleotide sugar metabolism (ko00520); the most abundant family of GHs for *C. cordycipiticola* was GH16 and GH18 (Table S9), and the most abundant family of CBM was CBM18s (Table S11) etc. 

Though abundant CAZymes were identified, the number of CAZymes in the genome of *C. cordycipiticola* (441) was much less than that of the other mycoparasitic fungi, *C. rosea* (656), *T. harziam* (516) and *T. atroviride* (473), especially those for cell wall degradation (Table 2). This gene loss may be attributed to its specialization as a parasite of *C. militaris*. In the genome of *E. weberi*, a specific parasite of ant cultivated *Leucoagaricus* spp., the depletion of CAZymes was also found [100]. There were fewer genes encoding cell wall-degrading enzymes and effector proteins in the genome of *Sclerotinia shiraiana* than those of *Sclerotinia sclerotiorum* and *B. cinerea*, which was probably a key factor of the narrow host range of *S. shiraiana* [88].

Fungal SMs serve survival functions by supporting the fungus in competition against other microbes, in self-protection and defense, and have important roles in communication and interaction with other organisms [103]. The number of SMs in *C. cordycipiticola* (66) was more than twice of its host *C. militaris* [18]. It was reported that compared with most other filamentous ascomycetes, the sequenced mycoparasitic *Trichoderma* species and *C. rosea* are especially enriched in SM-related genes [86] which serve in chemical warfare against their competitors and fungal preys and play a crucial role in mycoparasitism. SMs were also found to play a crucial role in specialized mycoparasite *E. weberi,* which kills its ant-cultivated host fungi from a distance, thereby likely involving secreted toxins [100]. The same phenomenon occurred during the infection of *C. cordycipiticola*. In some cases, for the hyphae of the host which did not contact with those of the pathogens directly, the cell organelles had been lost even if the cell wall was only partially degraded and deformed (Figure 3A–C). It was speculated that some diffusible SMs might play an important role during the infection process. Comparative genomic and transcriptomic analyses suggest that this fungus may produce coprogens, radicicol, depudecin-related and many other unknown compounds at the mycelial stage. Among them, coprogenss may be an important virulence factor of *C. cordycipiticola* and promote iron absorption during infection, which is our ongoing project.

The fruiting bodies of *C. militaris* have been consumed worldwide as a tonic [1]. Sometimes the mycelia of *C. cordycipiticola* may mix with the fruiting bodies for sale and so the safety is still of concerned. Our genome data suggest that there are some gene clusters for mycotoxin-production, such as solanapyrone, hypothemycin, zearalenone and citrinin-related compounds (Tables S14 and S15). Transcriptomic analysis revealed that at least solanapyrone and citrinin-related compounds are likely to be produced by this fungus at the mycelial stage because of the high or moderate expression of the encoding genes. From the safety point of view, the fruiting bodies which have been infected by *C. cordycipiticola* should not be consumed.

Phylogenomic analysis based on the single copy orthologs of 32 fungi revealed that *C. cordycipiticola* is evolutionarily close to its specific host, *C. militaris* (Figure 7), which makes it difficult to study the co-evolution. The divergence time estimation revealed that the branch of *C. militaris* diverged from *C. cordycipiticola* around 80.9–62.2 MYA (Figure 7) after or at the cretaceous extinction event (65 MYA), being concurrent with the Cretaceous diversification of fungal-arthropod symbioses supported by the oldest fossil evidence of animal parasitism by fungi [104]. Our analysis supported the conclusion that the shifts to mycoparasitism occurred several times during the evolution of hypocrealean fungi [105].

Mycoparasitism generally involves four sequential steps: chemotropism, recognition, attachment and cell wall penetration, and digestion of host cell content [106]. The present study gave little information on chemotropism and recognition, which are important for pathogenicity and disease control and will be our next project.

## Figures and Tables

**Table 1:** Genome feature comparison between *Calcarisporium cordycipiticola* and *Cordyceps militaris*.

Feature	*C. cordycipiticola* CGMCC 5.2193	*C. militaris* Cm01
Size (Mbp) *	34.51	32.2
Coverage (fold)	170×	147×
(G + C) percentage (%)	51.7	51.4
N50 (Mbp)	5.45	4.6
Percentage repeat rate	4.04	3.04
Protein-coding genes	10,443	9684
Average gene length (bp)	1692	1742
Percentage of secreted proteins (%)	9.02	8.00
Gene density (no. gene per Mbp)	302	257
tRNA genes	155	136
Pseudogenes	343	102
NCBI accession	PRJNA766243	AEVU00000000

* Mbp, mega base pairs.

**Table 2:** Comparison of CAZymes among fungi with different lifestyles.

Life-Style	Species	AA	CBM	CBM1	CE	GH	GT	PL	All
mycoparasitic fungi	*C. cordycipiticola*	67	26	4	56	211	71	6	441
	*C. rosea*	103	29	13	97	310	73	31	656
	*T. harzianum*	61	49	21	58	245	76	6	516
	*T. atroviride*	51	41	22	43	234	74	8	473
nematode parasitic fungi	*P. chlamydosporia*	63	33	9	43	278	86	7	519
	*H. minnesotensis*	54	28	0	32	137	80	2	333
entomopathogenic fungi	*B. bassiana*	51	30	3	33	160	79	2	358
	*C. militaris*	38	30	1	30	148	69	3	319
	*O. sinensis*	35	24	0	23	95	64	2	243
plant pathogenic fungus	*F. graminearum*	82	42	12	74	237	78	22	547
	*M. oryzae*	100	51	22	72	244	82	6	577
	*V. dahliae*	85	31	28	65	241	69	34	553
	*B. cinerea*	82	38	23	71	260	84	10	568

AA: Auxiliary activity family, CBM: Carbohydrate-binding module, CE: Carbohydrate esterase, GH: Glycoside hydrolase, GT: Glycosyltransferase, PL: Polysaccharide lyase.

**Table 3:** Secondary metabolite clusters in the genome of *Calcarisporium cordycipiticola*.

Feature	*Calcarisporium cordycipiticola* CGMCC 5.2193	*Calcarisporium arbuscula* NRRL 3705	*Calcarisporium* sp. 525	*Cordyceps militaris* Cm01
NRPS	14	12	17	8
T1PKS	18	23	24	6
Terpene	4	11	7	4
NRPS-like	12	0	0	6
PKS-NRPS-Hybrid (NRPS-PKS-Hybrid)	9	7	3	5
other	9	12	9	1
Total	66	65	60	30

## Data Availability

Publicly available datasets were analyzed in this study, which have been listed in Table S7.

## Supplementary Materials

The following are available online at https://www.mdpi.com/article/10.3390/jof7110918/s1, Figure S1. Large-scale cultivation of *Cordyceps militaris* and white mildew disease. Figure S2. A flow chart of the whole experimental design. Figure S3. Antibiotic sensitivity assays for *Calcarisporium cordycipiticola*. Figure S4. Optimization of ATMT in *Calcarisporium cordycipiticola*. Figure S5. Validation of transformants expressing RFP of *Calcarisporium cordycipiticola*. Figure S6. Interaction between Cordyceps *militaris* and *Calcarisporium cordycipiticola* in dual culture. Figure S7. Statistical analysis for high quality reads of *Calcarisporium cordycipiticola* genome sequencing data. Figure S8. *Calcarisporium cordycipiticola* mitochondrion genome. Figure S9. Evidence for gene prediction accuracy in *Calcarisporium cordycipiticola*. Figure S10. Histogram of KEGG distribution of predicted proteins of *Calcarisporium cordycipiticola*. Figure S11. Families of transposase genes and estimation of RIP. Figure S12. KEGG pathway enrichment analysis of the secreted proteins and putative pathogenic factors annotated by PHI database in *Calcarisporium cordycipiticola*. Figure S13. The putative pyranonigrins, depudecin and solanapyrone biosynthetic gene cluster of *Calcarisporium cordycipiticola*. Table S1. Characteristics of eight scaffolds. Table S2. The comparison of mitogenome between *Calcarisporium cordycipiticola* and *Cordyceps militaris*. Table S3. Characteristic of the annotated *Calcarisporium cordycipiticola*. Table S4. GO distribution of predicted proteins of *Calcarisporium cordycipiticola*. Table S5. KEGG category of predicted proteins of *Calcarisporium cordycipiticola*. Table S6. Repeat content of *Calcarisporium cordycipiticola* compared with other ascomycetes fungi Table S7. Species used in phylogenomic and evolution analyses. Table S8. The secretome, potential effectors, PHI genes and P450 genes in *Calcarisporium cordycipiticola*. Table S9. Family of glycoside hydrolases (GH) in *Calcarisporium cordycipiticola* compared with parasitic fungi Table S10. Sixteen GH18 genes in *Calcarisporium cordycipiticola*. Table S11. Family of carbohydrate-binding module (CBM) in *Calcarisporium cordycipiticola* compared with parasitic fungi. Table S12. Family of glycosyltransferases (GT) in *Calcarisporium cordycipiticola* compared with parasitic fungi. Table S13. Core genes of secondary metabolites (SMs) in *Calcarisporium cordycipiticola*. Table S14. Comparison of some clusters in *Calcarisporium cordycipiticola* with the related fungi. Table S15. Transcript analysis of genes of some secondary metabolite clusters. Table S16. Domain analysis of 38 PKS in *Calcarisporium cordycipiticola*. Table S17. Seventeen known mycotoxins were used for phylogenetic analysis.
