# Supplementary material for: Infection Process and Genome Assembly Provide Insights into the Pathogenic Mechanism of Destructive Mycoparasite Calcarisporium cordycipiticola with Host Specificity
Source: J Fungi (Basel). 2021 Oct 28;7(11):918. doi: 10.3390/jof7110918 (PMC8620734; doi:10.3390/jof7110918)

Figure S1. Large-scale cultivation of *Cordyceps militaris* and white mildew disease. **(A)** Large-scale cultivation of *C. militaris*. **(B-D)** Diseased fruiting bodies of *C. militaris*.

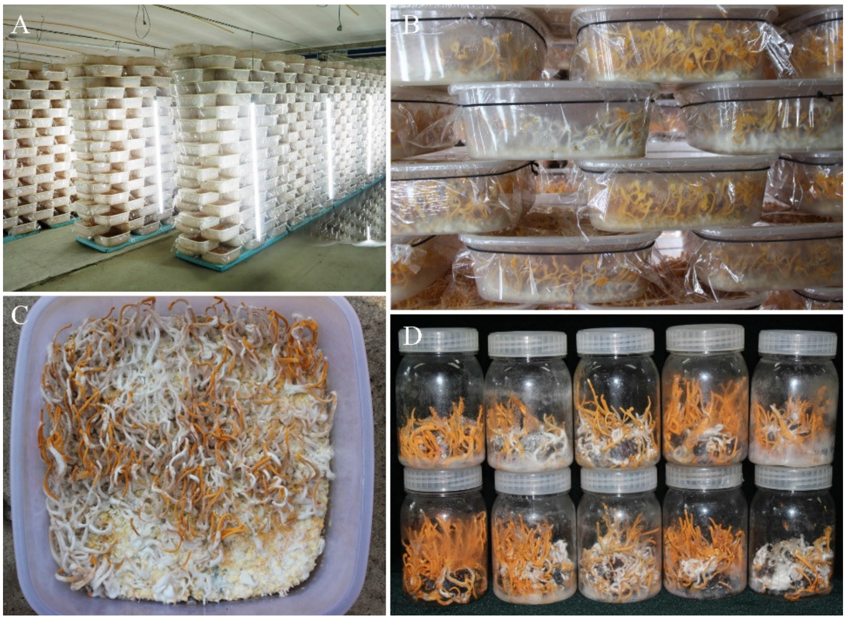

Figure S2. A flow chart of the whole experimental design.

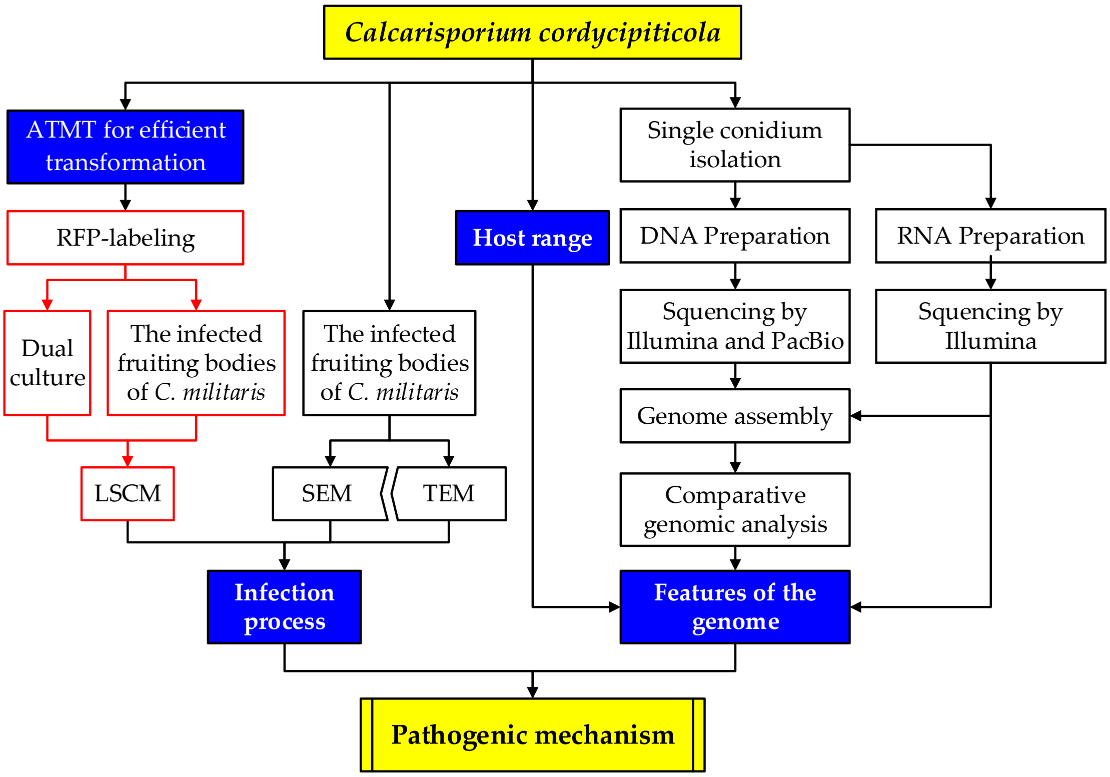

Figure S3. Antibiotic sensitivity assays for *Calcarisporium cordycipiticola*. **(A-D)** Different concentrations of geneticin, glufosinate-ammonium, bleomycin and hygromycin B.

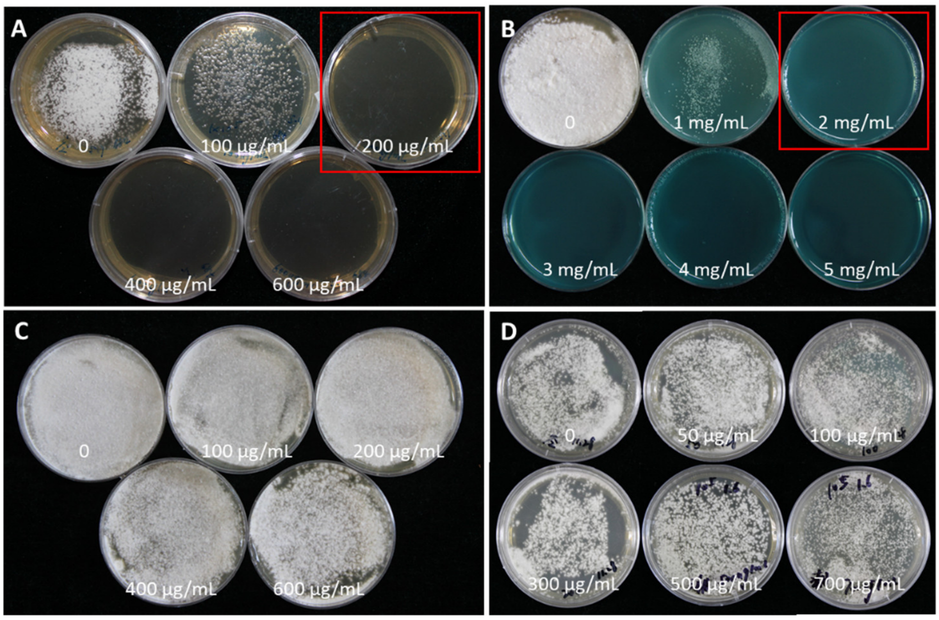

Figure S4. Optimization of ATMT in *Calcarisporium cordycipiticola*. **(A)** AS concentration was optimized under the condition of conidia density 10^6 conidia/mL, co-culture for 2 d and *A. tumefaciens* concentration of OD_{600} 0.6. **(B)** Days of co-culture was optimized under the condition of *A. tumefaciens* OD_{600} 0.8, conidia density 10^8 conidia/mL and AS concentration of 400 μM. **(C)** Conidia density was optimized under the condition of *A. tumefaciens* OD_{600} 0.8, co-culture for 2 d and AS concentration of 400 μM. **(D)** *A. tumefaciens* density was optimized under the condition of conidia density 10^7/ conidia/mL, co-culture for 3 d and AS concentration of 400 μM. All of the experiments were performed three times. The data are presented as means ± SD (n=3). *t*-test results are shown: *, $P < 0.05$; **, $P < 0.01$, ***; $P < 0.001$.

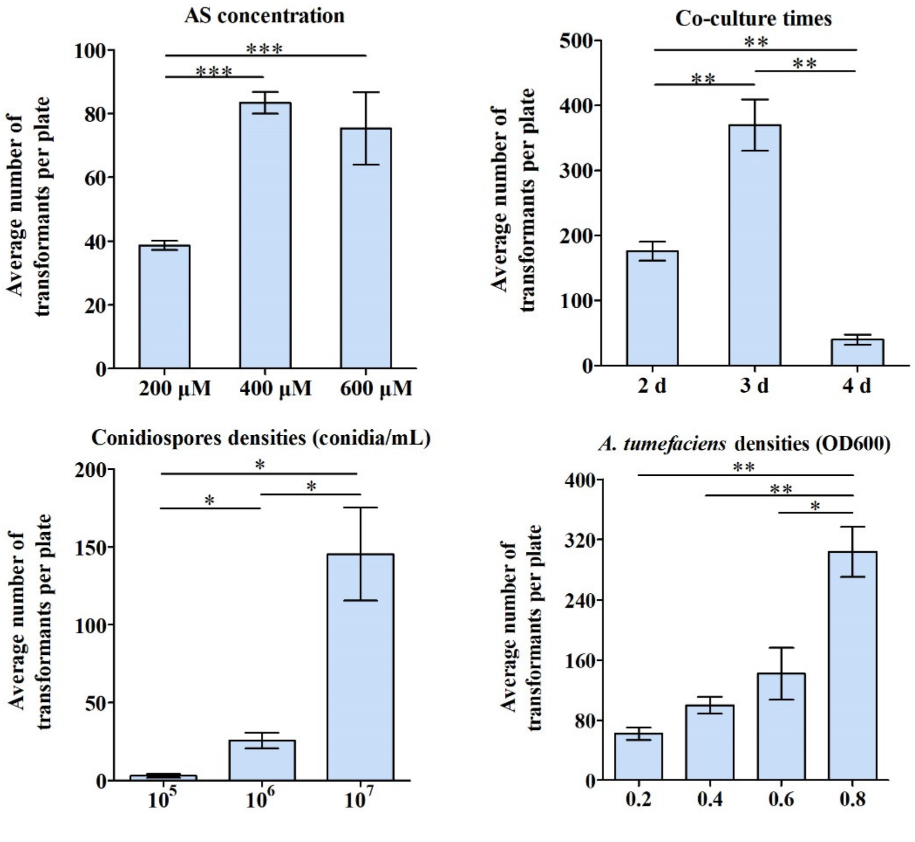

Figure S5. Validation of transformants expressing RFP of *Calcarisporium cordycipiticola*. **(A)** PCR validation. **1** Marker. **2-10** Transformants. **11** Plasmid pFGL815-neoR-tubCp-RFP. **12** Wild type of *C. cordycipiticola*. **(B)** Expression of the RFP in *C. cordycipiticola*. Scale bar =10 μm. DIC: different interference contrast.

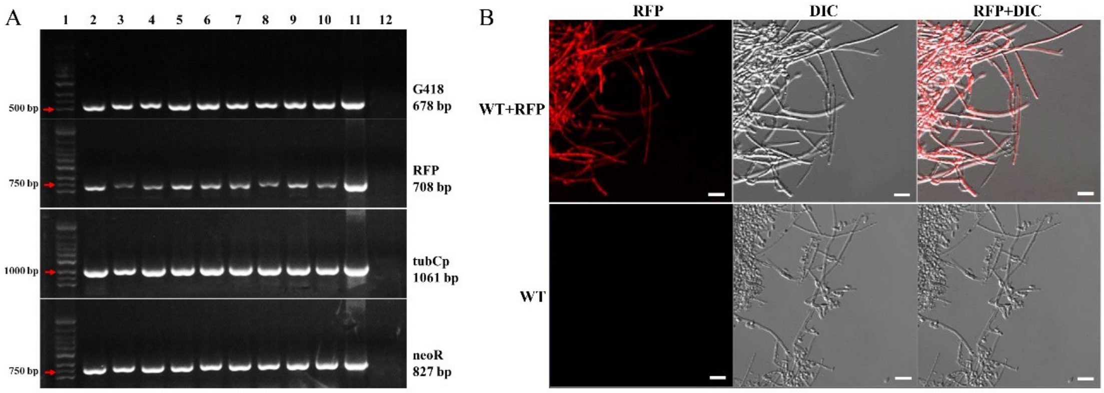

Figure S6. Interaction between *Cordyceps militaris* and *Calcarisporium cordycipiticola* in dual culture. **(A)** The hyphae of the touch point in dual culture observed by red fluorescent. **(B)** Overlay of fluorescent and brightfield micrographs. **(C)** Brightfield micrograph of B. **(D)** Dual culture of *C. militaris* and *C. cordycipiticola*. The inner illustration of B and C represented local enlargement. Scale bar = 2 cm in D and 10 μm in A-C.

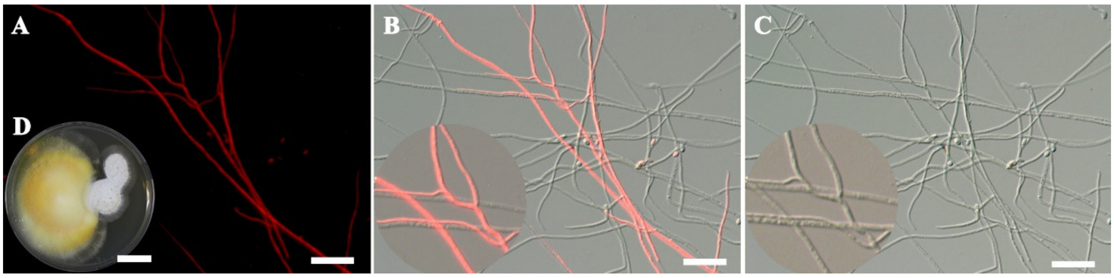

Figure S7. Statistical analysis for high quality reads of *Calcarisporium cordycipiticola* genome sequencing data. **(A)** K-mer distribution of Illumina NGS reads. The 400 bp library was subjected to paired-end 150 bp sequencing by Illumina HisSeq X10. The sequencing data (filtered reads: 5.50 Gbp; sequencing depth: 155×) were used to estimate the genome size, repeat content and heterozygosity. **(B)** Total of 6, 001, 867, 684 bp raw data and total of 5.93 Gbp clean data. 5, 933, 806, 642 reads with estimate the genome coverage 170× were obtained. Abscissa and vertical represent the subreads length, number of subread at specified length, respectively.

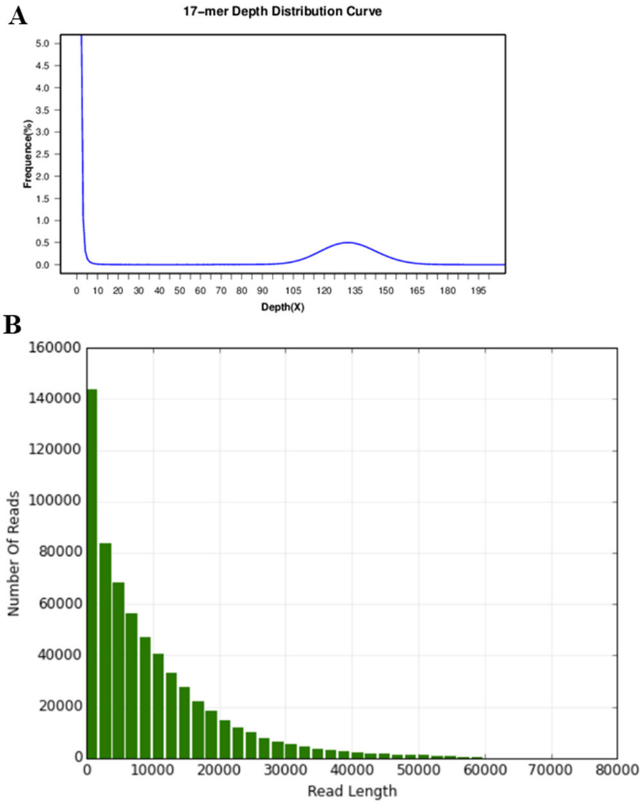

Figure S8. *Calcarisporium cordycipiticola* mitochondrion complete genome. Dark blue arrows represented tRNA genes. Red arrows represented rRNA genes. Wathet, purple and green arrows represented general coding protein genes.

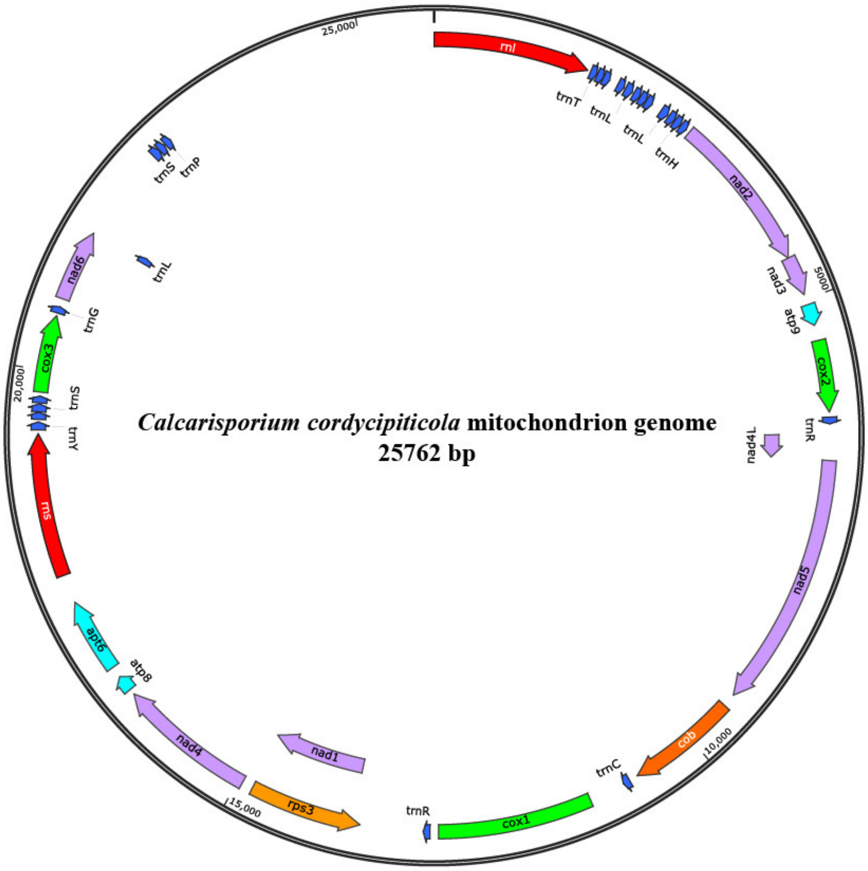

Figure S9. Evidence for gene prediction accuracy in *Calcarisporium cordycipiticola*. Three types of evidences were combined: functional annotation (at least one domain/motif), RNA-sequence support, orthologous genes between *C. cordycipiticola* and selected 10 fungi.

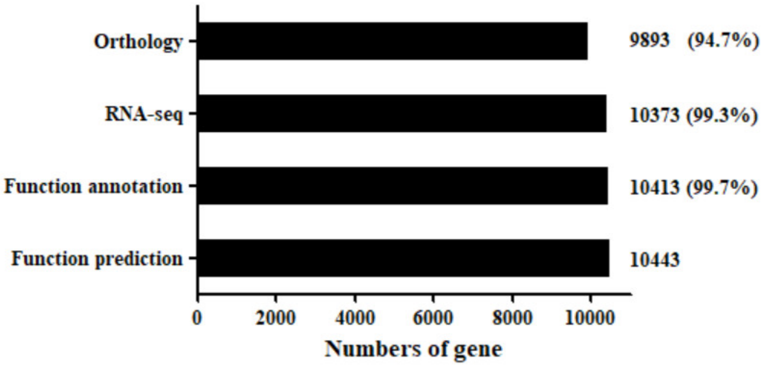

Figure S10. Histogram of KEGG distribution of predicted proteins of *Calcarisporium cordycipiticola*.

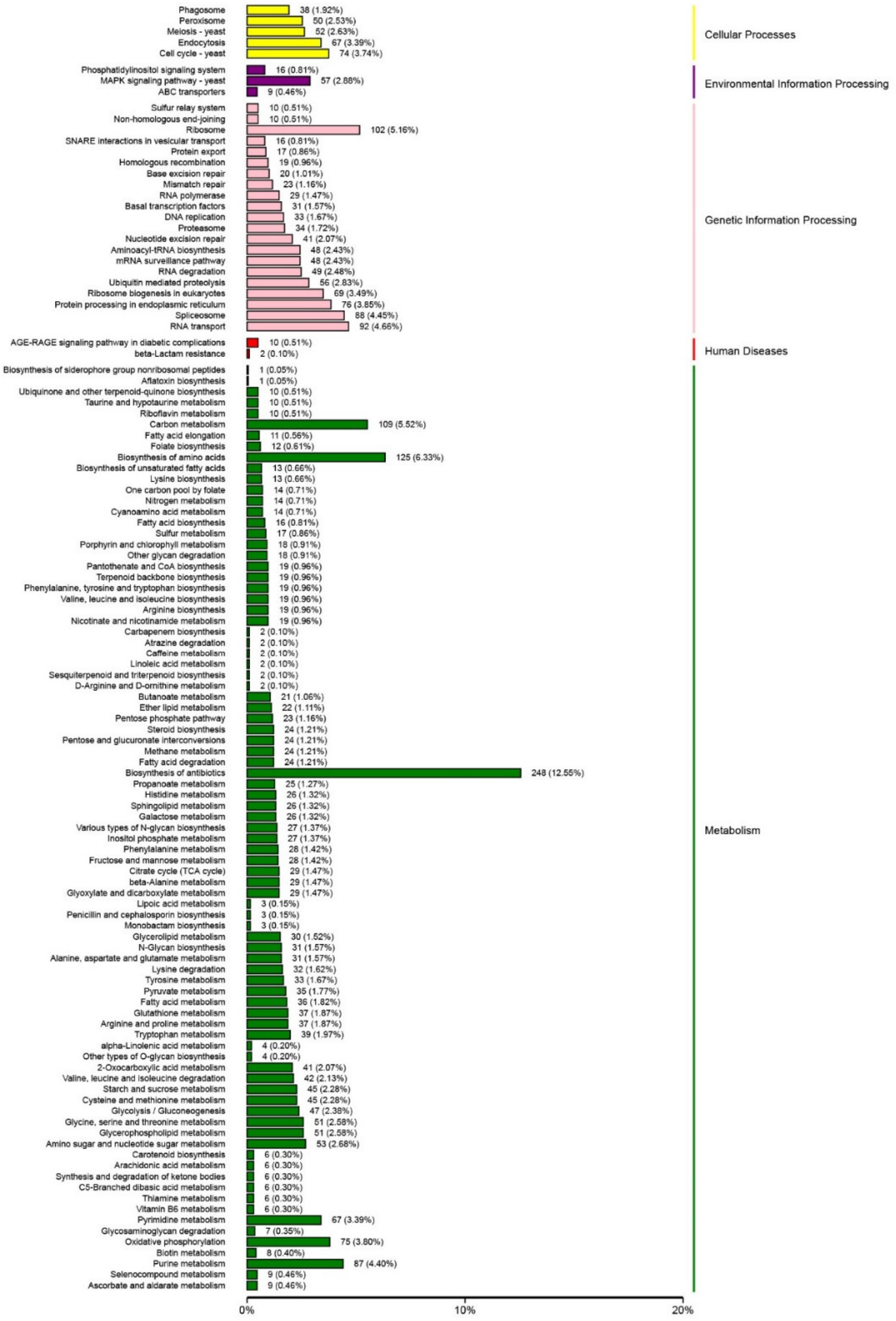

Figure S11. Families of transposase genes and estimation of RIP. (A) Families of transposase genes. (B) Estimation of RIP in *C. cordycipiticola*.

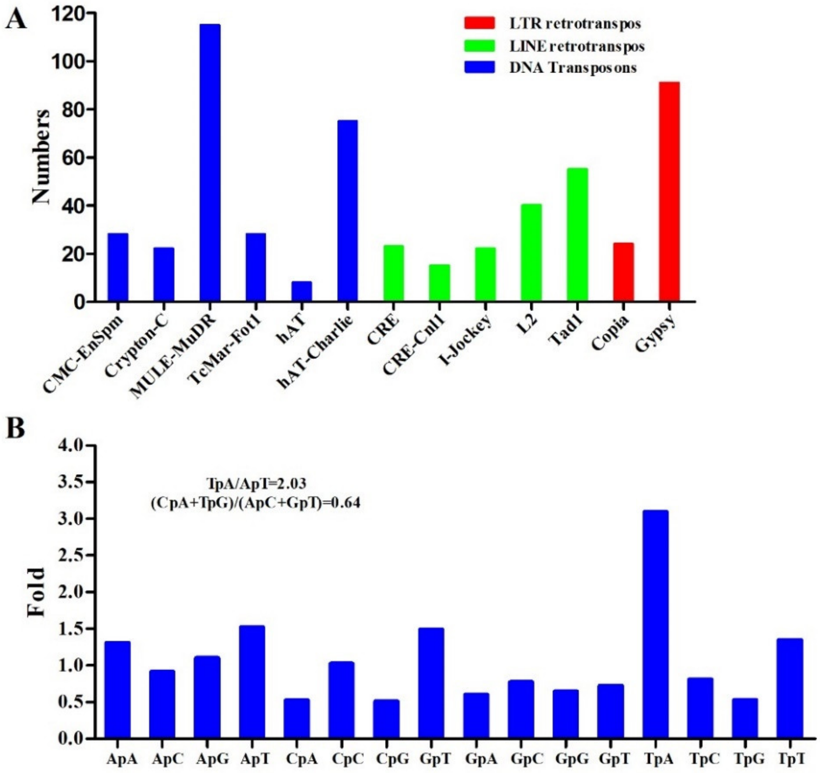

Figure S12. KEGG pathway enrichment analysis of the secreted proteins (A) and putative pathogenic factors annotated by PHI database (B) in *Calcarisporium cordycipiticola*.

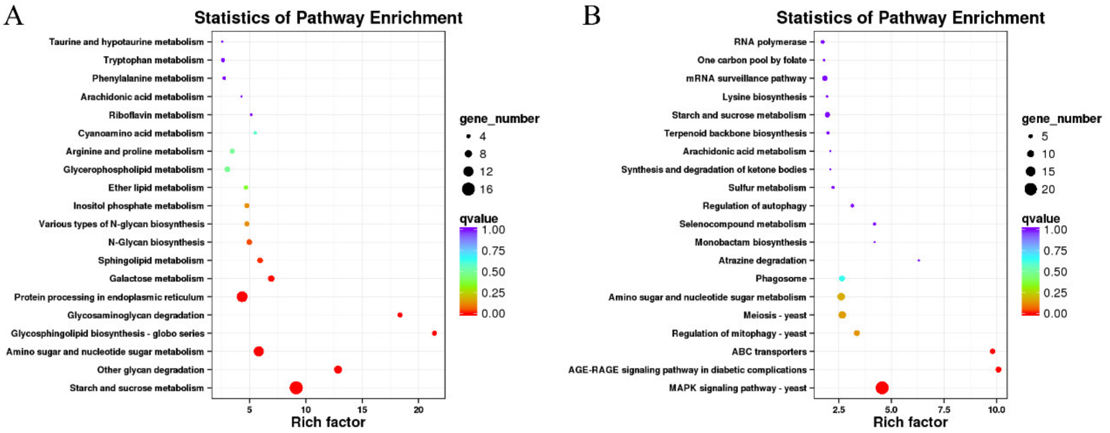

Figure S13. The putative pyranonigrins, depudecin and solanapyrone biosynthetic gene clusters of *Calcarisporium cordycipiticola* in this study. **(A)** Comparison of this cluster with the pyranonigrins cluster reported in *Aspergillus niger* CBS 513.88 and *Penicillium thymicola*. **(B)** Comparison with the depudecin cluster reportedin *Alternaria brassicicola* ATCC 96836. **(C)** Comparison with the solanapyrone cluster reported in *Alternaria solani* ATCC 16683. The identity of each homologue to *C. cordycipiticola* CGMCC 5.2193 counterpart is shown.

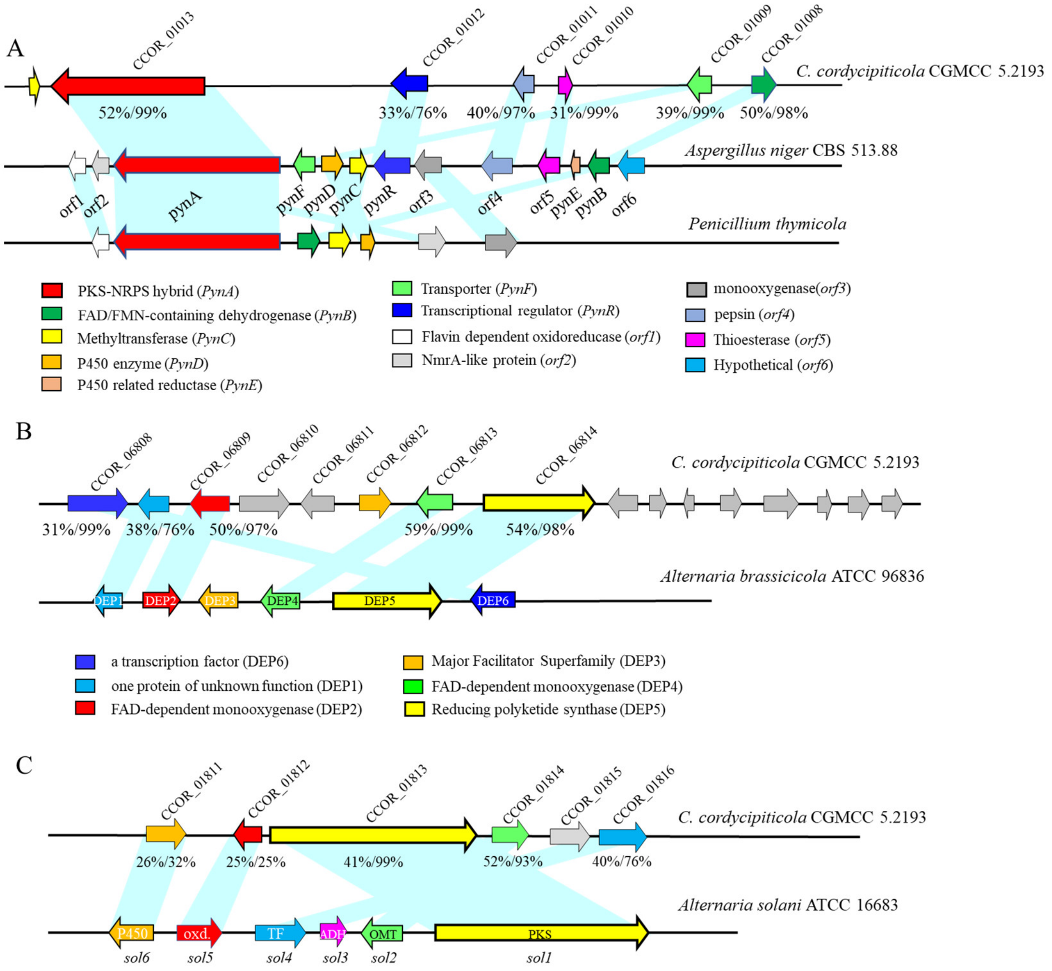

Supplement: Supplementary file 1 [file jof-07-00918-s001.zip › Supplemental Information 1.pdf]
